# “Boundary residues” between the folded RNA recognition motif and disordered RGG domains are critical for FUS–RNA binding

**DOI:** 10.1016/j.jbc.2023.105392

**Published:** 2023-10-27

**Authors:** Sangeetha Balasubramanian, Shovamayee Maharana, Anand Srivastava

**Affiliations:** 1 Molecular Biophysics Unit, Indian Institute of Science Bangalore, Bangalore, Karnataka, India; 2 Department of Molecular and Cell Biology, Indian Institute of Science Bangalore, Bangalore, Karnataka, India

**Keywords:** fused in sarcoma, RNA recognition motif, low-complexity domain, RNA–FUS binding, molecular simulations

## Abstract

Fused in sarcoma (FUS) is an abundant RNA-binding protein, which drives phase separation of cellular condensates and plays multiple roles in RNA regulation. The RNA-binding ability of FUS protein is crucial to its cellular function. Here, our molecular simulation study on the FUS–RNA complex provides atomic resolution insights into the observations from biochemical studies and also illuminates our understanding of molecular driving forces that mediate the structure, stability, and interaction of the RNA recognition motif (RRM) and RGG domains of FUS with a stem–loop junction RNA. We observe clear cooperativity and division of labor among the ordered (RRM) and disordered domains (RGG1 and RGG2) of FUS that leads to an organized and tighter RNA binding. Irrespective of the length of RGG2, the RGG2–RNA interaction is confined to the stem–loop junction and the proximal stem regions. On the other hand, the RGG1 interactions are primarily with the longer RNA stem. We find that the C terminus of RRM, which make up the “boundary residues” that connect the folded RRM with the long disordered RGG2 stretch of the protein, plays a critical role in FUS–RNA binding. Our study provides high-resolution molecular insights into the FUS–RNA interactions and forms the basis for understanding the molecular origins of full-length FUS interaction with RNA.

FET family of RNA-binding proteins (RBPs) aggregate in amyotrophic lateral sclerosis (ALS) and frontotemporal lobe degeneration (FTLD) (1, 2), two common neurodegenerative diseases usually affecting individuals over 50 years of age. Familial cases of ALS and FTLD contain point mutations (3, 4, 5) in the low-complexity regions of FET RBPs (6, 7, 8), which leads to disruption of RNA and protein homeostasis, a major pathogenic mechanism responsible for causing these diseases (9, 10, 11). RNA-binding interfaces of RBPs are low-complexity regions that are disordered in nature and impart structural flexibility or disorderliness, which is an integral part of biomolecular recognition in protein–protein or protein–nucleic acid complexes (12). Upon RNA binding, the low-complexity regions transition from disorder-to-ordered state (13). Fused in sarcoma (FUS) protein is one such multidomain protein in the FET family with self-association and RNA-binding properties (14, 15, 16). FUS plays a key role in RNA metabolism including splicing and transcription. Mutations in FUS cause dysregulation of RNA metabolism and cytoplasmic inclusion, a key event in FUS-associated ALS/FTLD pathogenesis (17).

Besides regulating RNA metabolism, FUS is also an important protein responsible for the formation of functionally important biomolecular condensates, which are membrane-less assemblies of biomolecules (proteins and protein–nucleic acid mixtures) formed because of their demixing from surrounding plasm by liquid–liquid phase separations and are now identified in all major compartments of cells—nucleus, cytoplasm, and mitochondria (18, 19). Nucleic acids and proteins containing disordered regions like FUS are mainly responsible for phase separation and condensate formation in cells. Importantly, proteins like FUS can form condensates by themselves or together with RNA and other low-complexity domain (LCD)–containing proteins in all the following three different systems—(i) *in vitro*, where individual components can be purified and added to form one or few component-based reconstituted condensates, (ii) *ex cellulo*, where purified scaffold proteins are added to the cellular extract to form complex condensates that are similarly complex to the cellular condensates, and (iii) *in cellulo*, these are the physiological condensates that often contain many different proteins and RNA.

Though FUS binds promiscuously with a wide variety of structured and unstructured RNA and DNA involved in transcription, splicing, and DNA repair and is present at high concentrations in the nucleus, yet only 1% of the total concentration is found in nuclear condensates. This phenomenon implies that the phase separation of FUS is dependent on RNA concentration, and accordingly, a high RNA–protein ratio, like seen in nucleus, is reported to prevent phase separation, whereas a low ratio of RNA and prion-like proteins, like seen in cytoplasm, promotes phase separation (20). Another study by Hamad *et al*. (21, 22) using fragments of promoter-associated noncoding RNA revealed RNA sequence–dependent regulation of FUS condensate formation. Together, it is clear that phase separation of FUS depends on the concentration of both specific and nonspecific RNA. Such an ambiguous behavior can only be facilitated by the conformational plasticity of the disordered regions of FUS making them adaptive to bind different RNAs. In general, the RNA sequence–dependent interaction and conformational changes in the aggregate-prone disordered regions of FUS protein are hypothesized to be responsible for the regulation of condensate or membrane-less organelle formation, although the exact molecular mechanism of this phenomenon is not well understood.

As shown in Figure 1*A*, FUS is a 526 amino acid (AA) long protein comprising a low-complexity region enriched with serine, tyrosine, glycine, and glutamine residues (SYGQ) at its N-terminal (1–165 AA), ordered RNA recognition motif (RRM, 281–377 AA), and zinc finger (419–454 AA) domains, separated by three Arg–Gly–Gly-rich RGG (RGG2: 378–418 AA; RGG3: 455–501 AA) domains. The region 166 to 269 AA can be further classified into a G-rich (166–222 AA) and RG/RGG-rich (223–268 AA) region; alternatively, the entire region from 166 to 269 is also called RGG1. The nuclear export signal (269–280 AA) and C-terminal PY-nuclear localization signal (502–526 AA) regions help in their cytoplasmic and nuclear localization (23). Several recent studies focused on molecular grammar behind phase-separating sequences have elucidated the importance of patterning and sequence arrangement (especially those of aromatic residues) toward the phase separation in intrinsically disordered proteins (IDPs) including FUS (23, 24, 25). An important outcome of our study is the important role played by residues sitting at the interface of folded and disordered regions of these proteins, which we call as “boundary residues” and which we discuss in detail in our article. This is quite interesting for FUS since there is an ambiguity in the literature in defining the boundary between RRM and RGG2 domains involving the residues 360-SGNPIKVSFATRRADFNR-377. Primarily, the N-terminal SYGQ domain (also called the LCD) is responsible for the phase separation and aggregation behavior of FUS. However, besides the N-terminal LCD, the FUS protein contains three RG/RGG-rich disordered regions with nucleic acid–binding ability that is also known to mediate phase separation (26, 27). Recently, intermolecular and intramolecular interactions between LCD and RGG regions have been identified as another driving force in stabilizing the FUS condensates (26).

**Figure 1:**
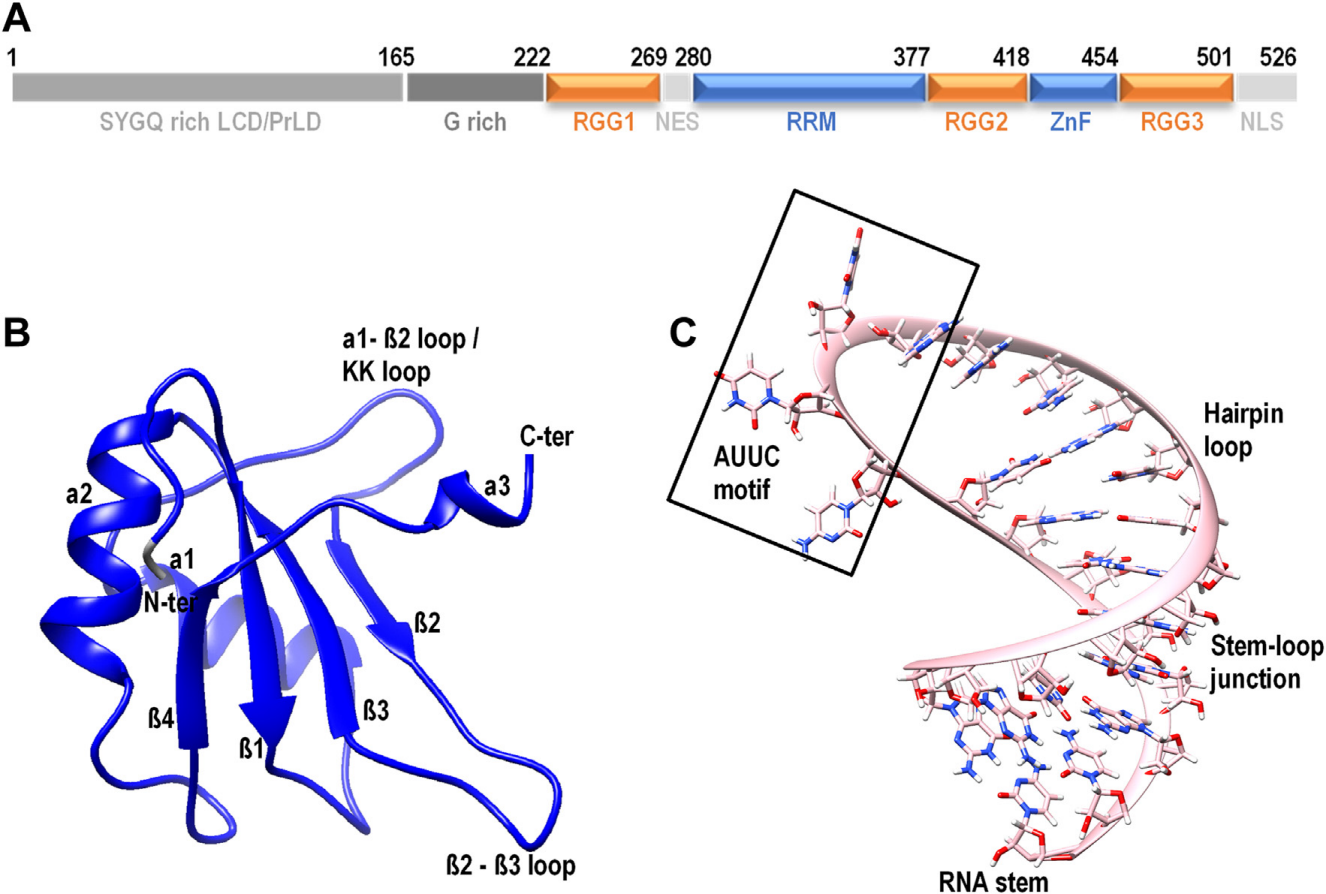
**Structure of FUS and target RNA.***A*, the domain organization of FUS. The RRM and ZnF (in *blue*) are the only folded domains, whereas the RGG (in *orange*) are disordered regions rich in RG/RGG motifs with nucleic acid–binding properties. The three-dimensional structure of (*B*) RRM domain and (*C*) RNA stem–loop structure with marked secondary structure motifs. FUS, fused in sarcoma; RRM, RNA recognition motif.

The RRM domain of FUS (28) is a folded domain, known to recognize several RNA as well as DNA targets in the genome, and multiple pieces of evidence exist for its recognition of a wide range of RNA and DNA structures (29, 30). The RRM domain comprises *β*1−*α*1−*β*2−*β*3−*α*2−*β*4 fold with a single short helical turn at the C terminus (structure shown in Fig. 1*B*). The RNA-binding pocket includes the surfaces of *β*-sheets 1, 2, and 3, the *α*1–*β*2 hairpin loop (also called KK loop) conserved in the FET family proteins, the *β*2–*β*3 loop, and the C-terminal helical turn. A recent advanced sampling study of RRM with a 12mer ssRNA has established the importance of loop dynamics for RNA binding (31). RNA recognition by FUS-RRM is mainly driven by the positively charged residues because of the lack of aromatic amino acids over the *β*-sheet surface (*β*3) and the longer *β*-hairpin connecting *α*1 and *β*2, which is unique and distinct from a canonical RRM (28). In a recent high-resolution molecular simulation study by Pokorná *et al*. (32), they focus on using all-atom molecular dynamics (MD) simulations to explore the properties of FUS–RNA complexes complementing the previous NMR studies. Several challenges and limitations in modeling an NMR faithful partial FUS–RNA complex were revealed in this study, and the authors identify the rich dynamics of FUS–RNA systems despite the addition of specific force field adjustments. However, the main observations from their study include the presence of coexisting substates of RNA, transient interaction of RGG with RNA minor groove, and a possible allosteric communication within the entire FUS–RNA complex. Another study by Sarthak *et al*. (33) addresses the effect of different force fields on FUS–RNA complexes, a well-known issue in MD simulation studies. This study tests the efficiency of conventional and modified force fields to sample FUS–RNA complexes and proposed that protein and RNA force fields that can share a four-point water model is optimal to sample the conformational dynamics of proteins like FUS having both structured and disordered regions. Despite the choice of force fields, this study has identified consistent interactions between RRM and RNA.

Several studies have identified sequence and structural motifs in RNA that are recognized by FUS (29, 34). The widely known RNA sequence motifs are GGUG, CGCGC, and GUGGU, whereas the structural motifs are an AU-rich stem–loop structure (Fig. 1*C*) (29) and a G-quadruplex structure (35). A recent NMR study by Loughlin *et al*. (23) has identified the structure of the RRM domain in complex with a stem–loop structured heterogeneous nuclear ribonucleoprotein (hnRNP) A2/B1 pre-mRNA (Fig. 1*C*). This study claims a shape specificity for the RRM domain and identifies a consensus motif of “NYNY” (N = Cyt/Ura/Ade/Gua; Y = Cyt/Ura) sequence in the single-stranded loop of the stem–loop RNA as the recognition motif. The ZnF domain is another ordered nucleic acid–binding domain in FUS, which shows specificity for a GGU motif. The NMR structure of the ZnF domain in complex with a 5mer RNA of sequence UGGUG has been solved by Loughlin *et al*. (23) to establish the binding mode and specificity of the ZnF domain. Together with the sequence specificity of the RRM domain, Loughlin *et al*. proposed the recognition of a bipartite motif in a stem–loop RNA (YNY and GG[U/G] within a 30 NT separation) by the RRM–RGG2–ZnF construct of FUS expressing both shape and sequence specificities.

The binding affinity of different domains of FUS with RNA has been identified previously by Schwartz *et al.* (27). This isothermal titration calorimetry study has shown that all FUS domains express weak binding affinity with RNA when present individually (27, 30). The binding affinity of wildtype FUS is 0.7 μM, whereas the two folded domains, RRM (*>*90 *μ*M) and ZnF (*>*175 *μ*M), show very weak affinity individually. Among the three disordered RGG regions, the RGG1 with 3 *μ*M is the strongest, followed by RGG3 with 9 *μ*M and RGG2 with 61 *μ*M. However, when the two weak binding domains RRM and RGG2 are present together, the binding affinity shows a drastic increase to 2.5 *μ*M. This is further enhanced to 1.9 *μ*M when RGG1 is also included. We assume that such a major jump in binding affinity among the individual (RRM and RGG2 with *>*90 *μ*M and 61 *μ*M, respectively) and combined RRM–RGG2 (2.5 *μ*M) constructs requires communication between the folded and disordered regions to bind RNA. Since the affinity data are biochemical in nature, structural binding geometry and interface behavior at the molecular scales are not apparent. Our study analyzes the interaction of the RRM domain with RNA and explores this hypothesis of a synergistic RNA-binding mechanism between RRM and RGG2 through all-atom MD simulations. Though the importance of FUS–RNA interaction has been well elucidated, the details of molecular interactions at the single-molecule level are still lacking. In this context, our study finds merit in exploring the characteristics of FUS–RNA interaction and also from the perspective of a varying number of RGG repeats. Also, given the recent findings establishing that the RGG regions interact with LCD in a condensate (25), our study can be used to further explore the possibility that there are RNA-mediated interactions between LCD and RGG in a condensate. Together, our study forms the basis for addressing an interesting mechanistic hypothesis regarding the RNA concentration–dependent phase behavior of FUS condensates.

The rest of the article is organized as follows. In the Results and discussion section, we highlight our salient findings. We find that the C-terminal helix in the RRM–RGG2 boundary region holds together the RRM–RNA complex, and the flanking RGG domains play a major role in enhancing RNA binding. We show the molecular-driving forces that explain how the number of repeats of RGG comes across as a major factor in the stable RNP complex formation. We also show how the sequence and length of the RNA are important in these complexes. Following the Results and discussion section, we describe our modeling and analysis methods in detail in the “Experimental procedures” section. Besides providing information on the molecular simulation protocols and reporting the systems under consideration, we also provide details about how we reconstructed these RNA–protein complexes with IDPs flanking on both sides of the folded RRM region. We have also used some ingenious approaches to analyze our complex trajectory data, and we also describe that in this section. The FUS–RNA complex systems modeled and simulated in this study are given in Table 1. We close the article with a short conclusion section. All our data including all trajectories and input files for our simulations, all analyses-related data, and codes are publicly available online.

**Table 1:** List of FUS–RNA complex systems studied in this work

Name	System description	Amino acids	Structure	Simulation (ns)
*FUS*_*RRM*_−*core*	RRM + 23mer RNA	276–368	6GBM	100 ∗ 1
*FUS*_*RRM*_	RRM + 23mer RNA	276–377	6GBM	1000 ∗ 3
*FUS*_*RRM*_−*KKK*_*mut*_	RRM + 23mer RNA	276–377	6GBM	1000 ∗ 1
*FUS*_380_	RRM–RGG2 + 23mer RNA	260–380	6SNJ	1000 ∗ 3
*FUS*_385_	RRM–RGG2 + 23mer RNA	260–385	6SNJ	1000 ∗ 3
*FUS*_390_	RRM–RGG2 + 23mer RNA	260–390	6SNJ	1000 ∗ 3
*FUS*_418_	RRM–RGG2 + 59mer RNA	260–418	Modeled	1000 ∗ 3
*FUS*_223−418_	RGG1-RRM–RGG2 + 59mer RNA	223–418	Modeled	1000 ∗ 3
*FUS*_390_−*RNA*_*mut*_	RRM–RGG2 + mut 23mer RNA	260–390	Modeled	1000 ∗ 1
*FUS*_418_−*RNA*_*mut*_	RRM–RGG2 + mut 59mer RNA	260–418	Modeled	1000 ∗ 1

## Results and discussion

### Boundary residues between the folded RRM and disordered RGG2 are critical for tight RNA binding

**Figure 2:**
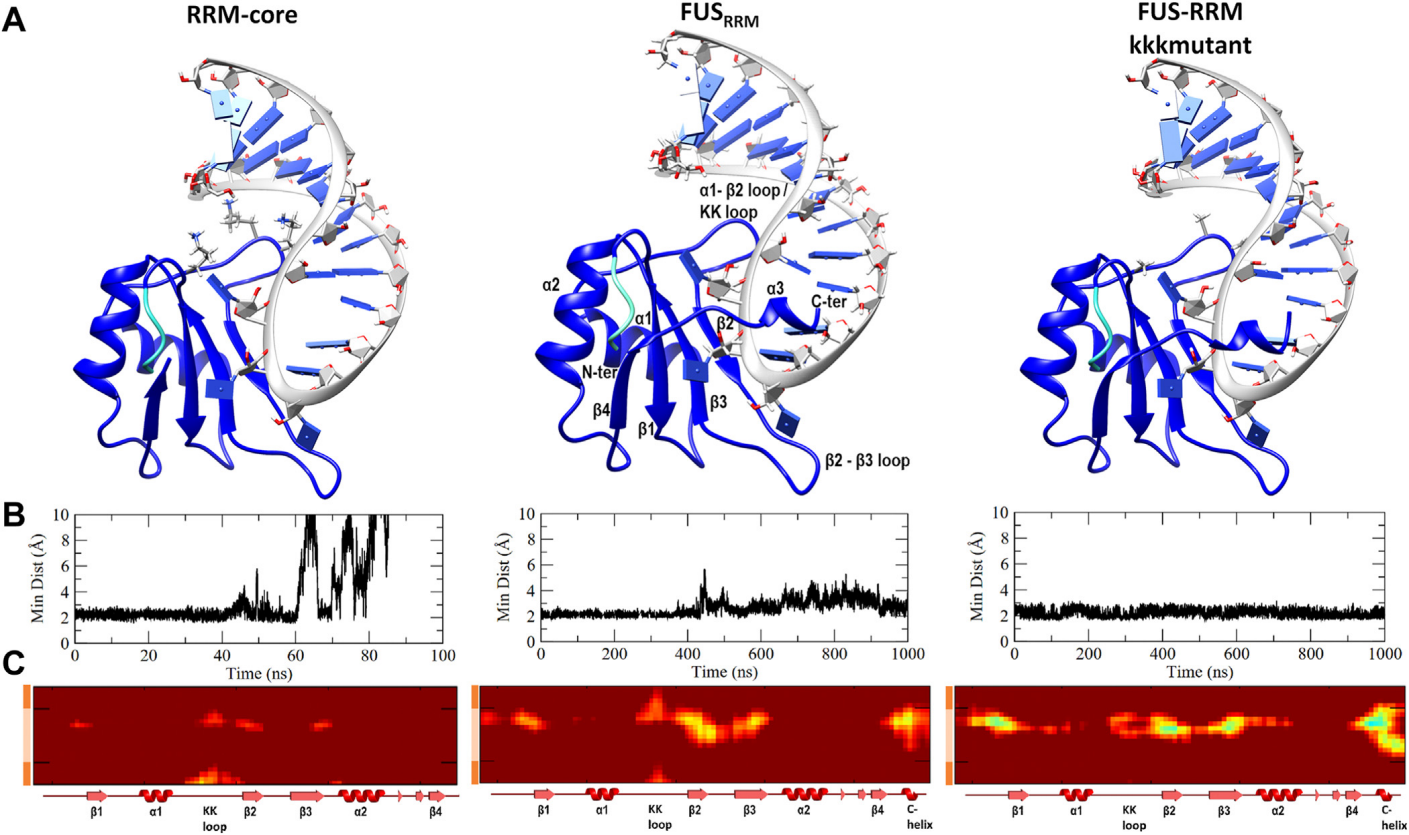
**Dynamics of RRM.***A*, the initial structures of RRM-core (276–368 AA), FUS_*RRM*_ (276–377 AA), and KK loop mutant (K312A/K315A/K316A). The NES residues 276 to 280 are colored in *cyan*. *B*, variation in the minimum distance between the β-sheet surface of RRM (286–290, 322–324, and 336–340 AA) and the RNA hairpin. *C*, interatomic distance matrix (in Å) between the residues of FUS RRM and RNA averaged over the last 100 ns simulation of the three replicates. The secondary structures of RRM are represented on the *x*-axis, whereas the RNA stem (*dark orange*) and RNA hairpin (*light orange*) are represented on the *y*-axis. AA, amino acid; FUS, fused in sarcoma; NES, nuclear export signal; RRM, RNA recognition motif.

An ambiguity exists in delineating the boundary between the RRM and RGG2 domains. Multiple reports regard this boundary to exist at different residues in the region 360 to 377 AA, with the majority placing it at 371 AA (23, 28, 36, 37, 38, 39). Consequently, we modeled a core RRM–RNA complex (276–368 AA), and the structure is shown in Figure 2*A*. During our simulations, the minimum distance between any pair of atoms in the β-sheet surface of RRM (286–290, 322–324, and 336–340 AA) and the RNA hairpin (Fig. 2*B*) remained within 2 Å for the initial 40 ns and starts fluctuating thereafter. After 60 ns, the minimum distance increases dramatically, indicating the dissociation of RNA from the core RRM (RRM_truncated.webm in the Supporting information). As depicted in Figure 2*C*, the interatomic distance matrix calculated as an average over the last 10 ns also demonstrates the dissociation of RNA from the core RRM. Liu *et al*. (28) previously reported a chemical shift perturbation for the residues 369 to 376 AA upon nucleic acid binding. The presence of these residues (369-ATRRADFNR-376 AA) significantly increases the volume of the RNA-binding pocket, as demonstrated by our CASTp-binding pocket analysis (Fig. S1). When the RRM comprises 276 to 377 AA, the volume of the binding pocket increases from 43.5 Å^3^ (for RRM 276–368 AA) to 1021 Å^3^. Hence, it is evident that the core RRM is insufficient to bind RNA and that the residues beyond 368 have a crucial function. Therefore, despite the ambiguity between studies, we consider the RRM domain boundary at 377 AA to be the minimal region required to bind RNA. In addition, the selection of 377 AA is consistent with previous studies including the NMR structure solution investigation by Loughlin *et al*. (23, 39), the structure used in our study. It is also significant to note that in the NMR solution structure, the boundary residues 369 to 377 AA form a single helical turn-like structure with six hydrogen bonds and two cation–*π* interactions with the RNA (shown in Fig. S2).

Following the RRM core, we simulated the RRM–RNA complex (Protein Data Bank [PDB] ID: 6GBM, 276–377 AA, Fig. 2*A*) in triplicates, for 1 μs per replica, and all three simulations show a similar unstable behavior. The individual all-atom RMSDs for RRM in all replicates are shown in the *bottom panel* of Fig. S3*A*. Our analyses show that the structure of RRM domain itself is stable with RMSD variations of less than 6 Å in all three replicates. By calculating the RMSD of the RNA while superposing the RRM, the nature of RNA binding with respect to the stable RRM domain can be obtained. This RMSD (*top panel* in Fig. S3*A*) indicates the stability of the binding orientation of RNA relative to RRM, and a substantial variation up to 25 Å indicates that the binding of RNA is dynamic and unstable in all the three replicates. The distance between the center of mass (com) of RRM and RNA was monitored and reported in the *upper panel* of Fig. S3*B*. Variations of ∼5 Å (22.5 ± 5 Å) suggest a weak/flexible RNA binding. Though the RMSD and com–com distances indicate that the RNA binding is unstable, the minimum distance between the RRM surface and RNA hairpin is less than 3 Å (averaged in Fig. 2*B* and *bottom panel* of Fig. S3*B*) indicating that the recognition motif remains in contact with the RRM. Even though one of the three replicates show fluctuations in this minimum distance, at the end of the 1 μs simulation, the distance reduces up to 2.5 Å indicating that the interaction of FUS with the recognition motif of RNA is regained. In order to identify the specific regions of RRM interacting significantly with the RNA, we plotted a matrix of distances between every pair of residues in RRM and RNA averaged over the last 100 ns. In Fig. S3*C*, the interatomic distances of the three replicates are shown, and these distances decrease on a red to blue scale with brighter intensities representing tighter binding. Though we observe widely different dynamics of RNA, we also note from the average of all three replicates in Figure 2*C* that the distances between the RNA hairpin and specific regions of FUS like the *β*-sheet surfaces, KK loop, and C-terminal helix are well preserved with the NMR structure (Fig. S3*C*).

Structurally, the interaction of RNA with RRM can be classified based on the interacting regions as (i) the surfaces of *β*-strands 1, 2, and 3 with the recognition motif AUUC, (ii) the *β*2–*β*3 loop with the AUUC motif, (iii) the KK loop with the major groove of the stem–loop junction, and (iv) the C-terminal helical turn with the RNA backbone (Fig. 1). The superposition of the RRM–RNA complex before and after 1 μs simulation reveals the unwinding of the C-terminal helix and its displacement from the initial position, resulting in the loss of interactions with the RNA backbone (Fig. S3*D*). Consequently, the repositioning of RNA also disrupts interactions with the other regions of FUS. The interaction of the AUUC recognition motif with various regions of FUS was monitored, and the interacting residues with the lifetime of each interaction are listed in Table S1. Several of the interactions present in the NMR structure are retained in the FUS_RRM_ even after simulations, albeit the lifetimes of few of these interactions like Arg328 and Arg372 are lesser. Particularly, the interactions of adenine and cytosine at the first and fourth positions of the recognition motif are lesser and varies from the NMR structure indicating a change in binding orientation of the RNA relative to the RRM. We report the variations in Table S1 indicating deviations in binding orientation of the RNA relative to the RRM. Even though the residues Phe288, Arg328, and Lys334 expressed *π–*stacking or *π*–cation interactions with the RNA bases, the interacting pairs from the initial complex do not have very tight interactions. Altogether, the RNA binds weakly with the RRM domain even though the RNA motif AUUC demonstrates multiple strong contacts with the *β*-sheets of the RRM domain. Particularly, the boundary residues making up the C-terminal helix serve a crucial role in retaining the RNA close to the RRM domain.

The Lys residues in the KK loop (312, 315, and 316 AA) are believed to be essential for RNA binding and subcellular localization. Moreover, mutational studies on the KK loop revealed similar chemical shifts for the mutant RRM–RNA/DNA complex and mutant-apo RRM, indicating that the mutation impairs nucleic acid binding (28). However, our simulations indicate that the boundary residues between the RRM and RGG2 also play a crucial role. In addition to the well-known KK loop, this previously unexplored C-terminal region of RRM (369–377 AA) serves an important role in RNA stabilization. The importance of this C-terminal region for RNA binding has been vastly overlooked to date. Despite the fact that NMR studies have identified their involvement in RNA binding through NMR chemical shift changes (28), only the KK loop has been primarily attributed to the RNA-binding property, as it is unique to FUS-RRM. In order to understand the significance of the KK loop for RNA binding, we modeled an RRM–RNA complex with KK loop mutations (K312A/K315A/K316A) as shown in Figure 2*A*. The minimum distance between the β-sheet surface of RRM and the RNA hairpin during the 1 μs simulation of the mutant RRM–RNA complex shows no total dissociation of RNA (Fig. 2*B*). Though there was no dissociation, the interatomic distance matrix reveals a distinct pattern of RRM–RNA interaction when compared with the FUS_*RRM*_ complex (Fig. 2*C*). While the distance between the KK loop and RNA stem increases, the distance between the KK loop and the RNA hairpin loop decreases indicating a rearrangement of the RNA. Based on these results where we witness severe rearrangements in the RNA binding pose leading to weaker binding, we hypothesize that the KK-loop mutation prevents initial recognition and binding of RNA or DNA. This is consistent with the experimental observation that mutations in the unique KK loop of FUS-RRM impair or drastically reduce the affinity for nucleic acid binding (28).

Because of the absence of stacking interactions between FUS-RRM and RNA, it has been reported previously that the stability is driven by electrostatic interactions, with the KK loop playing a major role. However, our study shows that these electrostatic interactions are insufficient to fully stabilize the RNA in the absence of 369 to 377 AA. These two regions are positioned to interact with RNA from opposing sides, and together, they bind both the grooves of the RNA stem–loop structure. The importance of this C-terminal region is further highlighted by the fact that they form the boundary between RRM and RGG2, and the interaction of RGG2 with RNA depends on the spatial arrangement of the boundary residues. According to biochemical investigations by Schwartz *et al.* (27), the presence of RGG2 increases the RNA-binding affinity of FUS, and the affinity also depends on the number of RGG repeats present. Hence, we further extended our study to include RGG repeats of varying lengths and explore their significance in enhancing the RNA-binding affinity.

### Electrostatically dominant RGG2–RNA interaction is modulated by the number of RGG repeats

**Figure 3:**
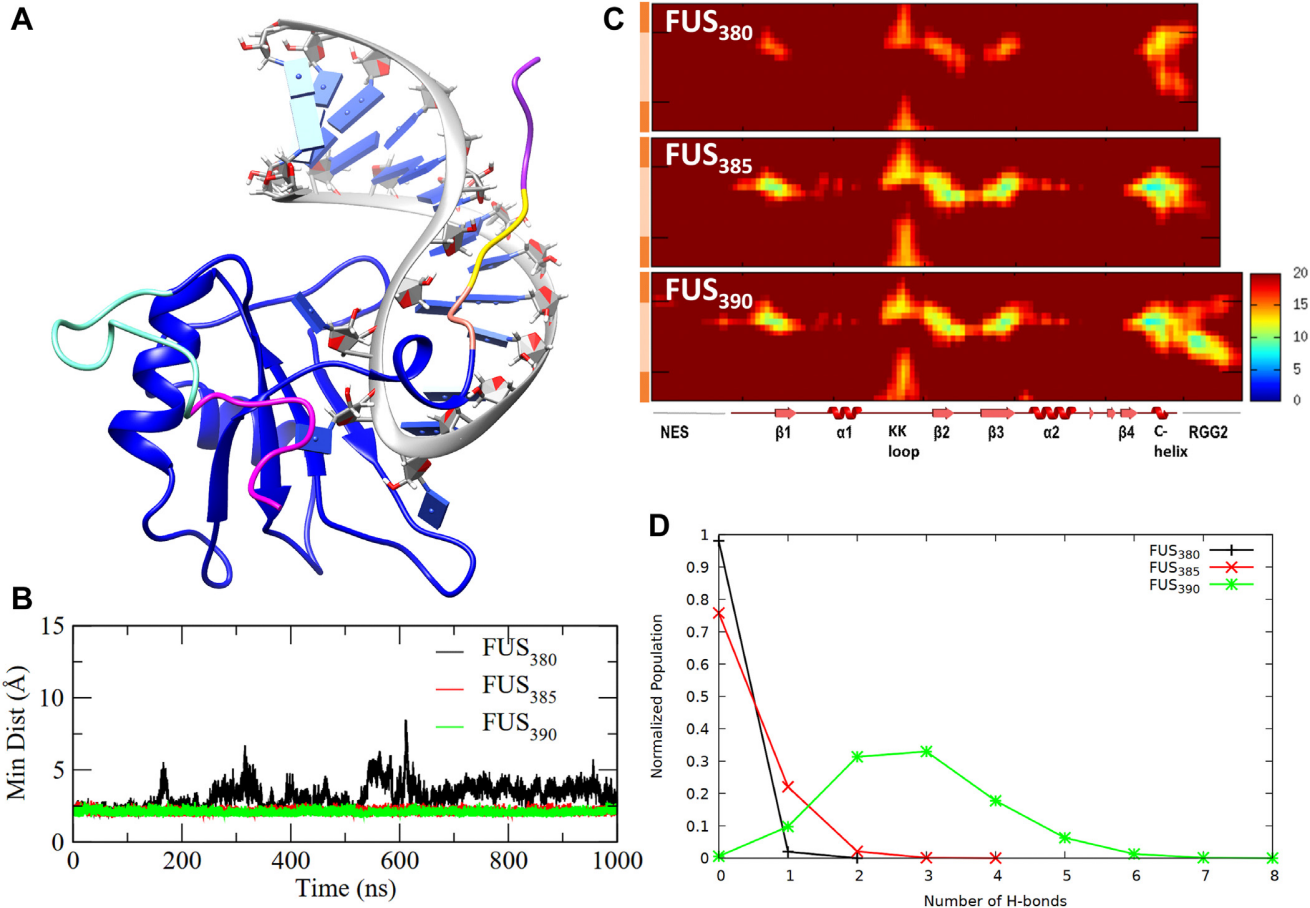
**Dynamics of FUS**_**380**_**, FUS**_**385,**_**and FUS**_**390**_**.***A*, the structure of FUS_390_ was used to model the truncated structures differentiated as 378 to 380 AA in *pink*, 381 to 385 AA in *yellow*, and 386 to 390 AA in *purple* colors. *B*, average variation in the minimum distance between the β-sheet surface of RRM (286–290, 322–324, and 336–340 AA) and the RNA hairpin of the three independent simulations. *C*, interatomic distances (in Å) between the residues of FUS and RNA averaged over the last 100 ns of the three replicate simulations. The same matrix for independent runs is shown in S4 in Supporting information. The secondary structures of FUS are represented on the *x*-axis, whereas the RNA stem (*dark orange*) and RNA loop (*light orange*) are represented on the *y*-axis. *D*, population distribution of the number of hydrogen bonds formed between the RGG2 and RNA. AA, amino acid; FUS, fused in sarcoma; RRM, RNA recognition motif.

The RGG2 spans residues 378 to 418 and contains five RGG repeats. A previous biochemical study by Schwartz *et al.* (27) determined that a minimum of three RGG repeats are required to increase RRM–RNA affinity closer to the wildtype range, and that the addition of further RGG repeats only slightly enhanced the binding affinity. To investigate the molecular basis for this observation, we simulated RRM–RGG2–RNA complexes with a varying number of RGG repeats (listed in Table 1) and analyzed their interactions with RNA. Initially, the function of the first three RGG repeats (up to 390 AA) was investigated, as the binding affinity increases dramatically only after the addition of the third repeat. Accordingly, the coordinates of RGG2 (PDB ID: 6SNJ shown in Fig. 3*A*) were truncated at 380, 385, or 390, respectively, to model three different complexes with variable numbers of RGG repeats. Each of these three complexes were simulated for 1 μs in triplicates (total 3 μs run for each complex). The distance between the β-sheet surface of RRM domain and the RNA hairpin in RRM–RGG2–RNA complexes containing one (*FUS*_380_), two (*FUS*_385_), and three (*FUS*_390_) RGG repeats is shown in Figure 3*B*. The data in Figure 3*B* report the average from the three replicas, and we show the individual replica data in Fig. S4*A*. A minimum distance of *<*3 Å indicates that the RNA continues to interact with the RRM domain. As seen from the figure, RNA complexed with *FUS*_385_ and *FUS*_390_ have tighter binding than *FUS*_380._ Minimum distance plots between the β-sheet surface of RRM domain and the RNA hairpin show the same trends for the three replicas as well.

The FUS–RNA interactions were analyzed further in detail to understand the interaction by different regions of FUS and the effect of the number of RGG repeats on binding affinities. The interatomic distance matrices between the RRM and RNA, averaged over the last 100 ns of the triplicates for the three systems, are shown in Figure 3*C*, and we show the data for the individual trajectories in Fig. S4*B*. The analyses clearly illustrate the difference caused by varying the number of RGG repeats. In *FUS*_380_, the distance between RNA and RRM increases, as indicated by the general decrease in RNA intensities. Nevertheless, the C terminus of RRM and RGG2 (370–380 AA) remains close to the RNA. On the other hand, the interatomic distances between RRM and RNA in *FUS*_385_ decrease significantly as evidenced by the strong intensities of RNA hairpin with the KK loop as well as the *β*-sheets. Notably, residues 370 to 380 remain securely bound to the RNA, whereas the residues 381 to 385 do not express any significant association with the RNA. Upon extending the RGG2 to include the third RGG repeat, in *FUS*_390_, the residues 370 to 390 are closer to the RNA hairpin and stem–loop junction. Similarly, the number of H-bonds between RGG2 and RNA increases proportionally to the number of RGG repeats. The number of H-bonds between RGG2 and RNA was calculated from the three independent simulations (Fig. S4*C*)), and a collective histogram is plotted in Figure 3*D* for all the three systems. The *FUS*_380_ complex shows a maximum of two H-bonds, whereas the *FUS*_385_ complex shows two additional H-bonds. Interestingly, *FUS*_390_ complex expresses the highest number of H-bonds (∼8) between RGG2 and RNA highlighting a significant shift in the interaction pattern with the addition of only one more RGG repeat.

As we have shown in previous sections, the C-terminal helix serves a crucial role in RNA stability. Visual analysis of the trajectories also reveals interesting changes in the stability of this C-terminal helix and consequent changes in RNA stability. Hence, we performed secondary structure analysis on the trajectory data. Fig. S5 shows that the C-terminal helix is highly disrupted in *FUS*_380_, and its stability increases with increasing lengths of RGG2. The stability of this C-terminal helix has a significant impact on the stability of RNA binding as evidenced by *FUS*_380_ (run2 data in Fig. S5). In spite of the RGG2 being shorter, the C-terminal helix is retained, and thereby, the RNA also remains close to the RRM. Fig. S6 depicts the RNA–protein complex structures after 1 μs of simulation superimposed over the respective reference structure. For each of the three systems, we show the conformation for all three repeats in the Supporting information. Similar to *FUS*_*RRM*_, the RGG2 in *FUS*_380_ is insufficient to stabilize the RNA when the C-terminal helix is absent, whereas in *FUS*_385_, the RGG2 remains coiled near the AUUC motif of the RNA. Interestingly, RGG2 remains bound to the RNA spine in *FUS*_390_. The structure of RNA in *FUS*_380_ is highly distorted, and the RNA reorients in the binding pocket, which explains the loss of intensities observed in the interatomic distance matrix. Interestingly, the overall RNA structure of both *FUS*_385_ and *FUS*_390_ is preserved.

The interaction of the AUUC motif with the RRM domain was monitored in the three systems, and the interactions are shown in Table S1. With the exception of a *π-*interaction with Arg328 and hydrophobic interactions with Tyr325 and Arg372, the RNA in *FUS*_380_ does not express any other interactions with the RRM, which reinforces the weak intensities in the interatomic distance matrix. Contrarily, the RNA in *FUS*_385_ exhibits several novel interactions with RRM, such as *π*-interactions with Thr286, Arg372, and Phe375, H-bond interactions with Tyr325, Thr338, and Arg371, and other hydrophobic interactions. In addition to the interactions present in the NMR complex, the *FUS*_390_ complex shows additional interactions, indicating a tighter RNA binding.

In order to understand the contribution of various residues in *FUS*_380_, *FUS*_385_, and *FUS*_390_ that interact with each RNA base, we monitored the number of interactions expressed by each amino acid (sum of electrostatic, hydrophobic, and hydrogen bonds), and we present the data as a histogram (Fig. S7). Also, to collectively understand the FUS–RNA interactions in the three independent simulations of each system, the histograms in Fig. S7 were averaged over the final 100 ns of all three replicates. According to Fig. S7, RRM domain mediates the majority of FUS–RNA interactions, whereas the RGG2 contributes only a few interactions to RNA binding. In addition, Arg as well as Lys residues dominate the interactions between RRM and the RNA hairpin. Furthermore, Asp and Phe residues show several interactions over the length of RNA, whereas other residues like Thr, Ala, Glu, and Gly also express a few interactions. The RNA-binding pocket in RRM is lined with three lysines from the KK loop, one arginine from the *β*2–*β*3 loop, and two arginines from the C-terminal helix, whereas the RNA-binding pocket lacks Asp, apart from one in the C-terminal loop (see Fig. S8 that we provide as a visual aid to highlight the key residues). The RNA hairpin in *FUS*_380_ expresses several interactions with Arg, Asp, and Lys residues of RRM suggesting a unique and distinct RNA-binding mode. Also, the single Arg residue in RGG2 interacts with multiple RNA bases at the stem–loop junction, indicating that the RNA is extremely dynamic. It is noteworthy that the interaction pattern of RRM with RNA in *FUS*_385_ and *FUS*_390_ is very similar except for those involving Arg residues. The Arg in the RRM domain is solely responsible for the contacts with the AUUC motif in *FUS*_385_. Whereas in *FUS*_390_, the Arg from both RRM and RGG2 are involved in binding the RNA hairpin and stem–loop junction. All three Arg residues of RGG2 in *FUS*_390_ interact with the stem–loop junction of RNA, which is quite intriguing. This observation demonstrates that the addition of RGG repeat provides multiple interaction sites for RNA and that both RRM and RGG2 play a role in stabilizing the RNA. The first RGG repeat is located close to the C-terminal helix in a position that is structurally constrained to provide any stability to the RNA. Moreover, visualizing the simulation trajectory of *FUS*_380_ also revealed that the loss of helicity at the boundary affects their interaction with RNA. Further extension of RGG repeats as in *FUS*_390_ stabilizes this helix and mediates their interaction with the RNA.

**Figure 4:**
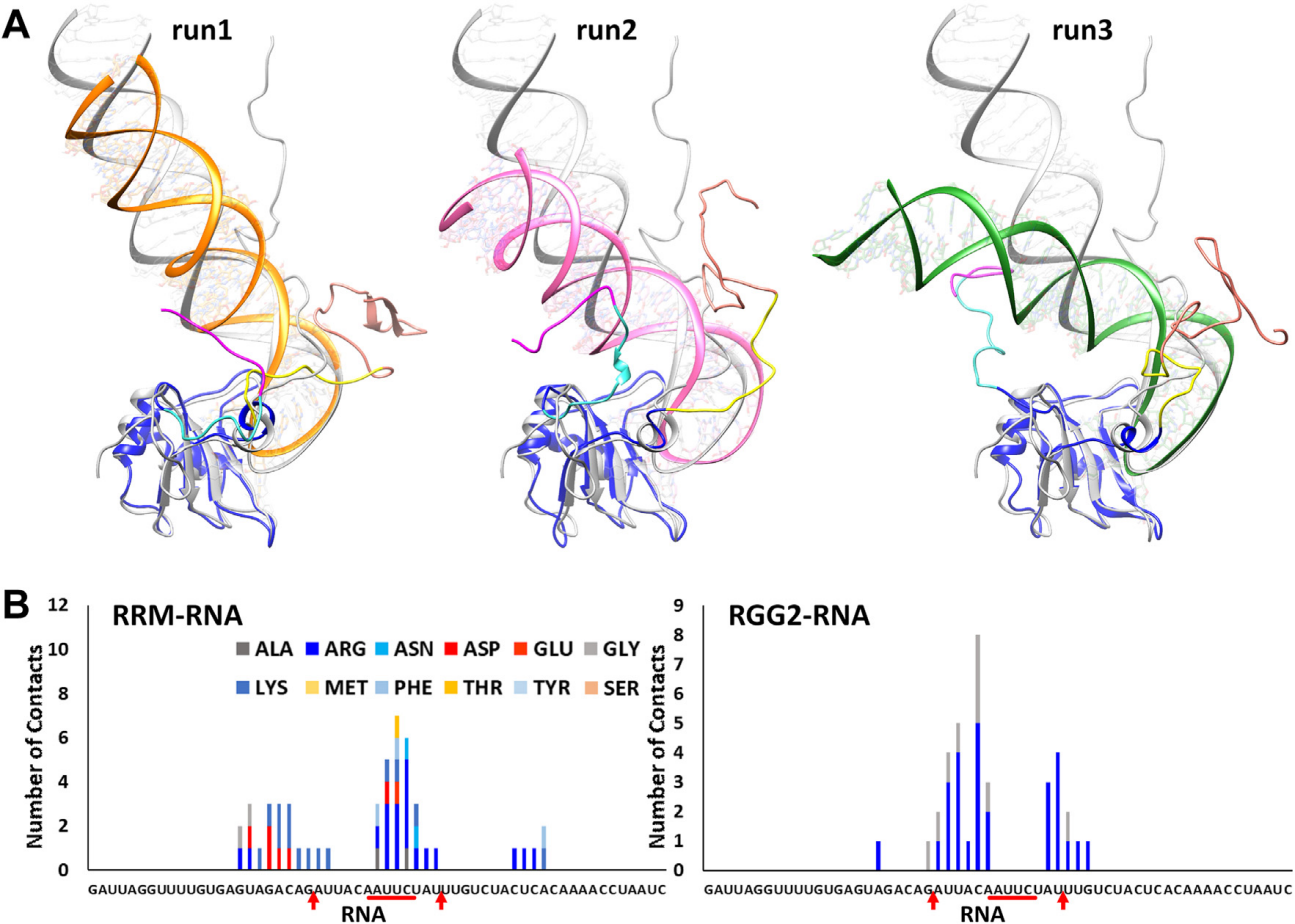
**Dynamics of FUS**_**418**_**.***A*, superposition of the modeled FUS_418_–RNA complex in *gray* over the 1 μs simulated conformation of the three replicates. The different regions of FUS are colored as RGG1 in *magenta*, NES in *cyan*, RRM in *blue*, and the three simulated RNAs in *orange*, *pink*, and *green* colors, respectively. The RGG2 up to 390 (*yellow*) is colored distinctly from 390 to 418 (*salmon*) to highlight the importance of this region. Amino acid–wise interactions depicting the number of interactions by each amino acid in the (*B*) RRM and RGG2 domains with the individual bases of the 59mer RNA. The stem–loop junctions and the recognition motif are marked on the *x*-axis. FUS, fused in sarcoma; NES, nuclear export signal; RRM, RNA recognition motif.

The RRM domain has five arginines, three of which line the RNA-binding pocket. On the other hand, RGG2 (377–418 AA) is compositionally biased and contributes additional five arginines with 28 glycines. The significance of the addition of one RGG repeat in *FUS*_390_–RNA complex relative to the *FUS*_380_ or *FUS*_385_ complexes was established previously. Hence, we sought to investigate the reported increase in RNA-binding affinity caused by the addition of two arginines and ∼20 glycines (391–418 AA) to *FUS*_390_. The compositional bias of RGG2 is such that, three of the five RGG repeats are located within the first 13 AA, whereas ∼70% of the following stretch, 391 to 418 AA is made of Gly and only two Arg. Therefore, we propose to further classify the RGG2 into an RGG-rich region (378–390 AA) and a G-rich region (391–418 AA). The *FUS*_418_–RNA complex was modeled as a highly disordered structure containing all five RGG2 repeats. The modeling was performed by the sequential addition of three to five residues with 50 ns of restrained simulations at each step. In order to accommodate the extended structure, the length of the double-stranded stem of RNA was also extended by adding 10 base pairs. The modeling protocol is described in detail in the Experimental procedures section, and Figure 4*A* depicts the structure of the modeled system as well as the 1 μs simulated RRM–RGG2 construct. Similar to the other previous systems, the residue-specific interaction histogram for the *FUS*_418_–RNA complex was calculated and shown in Figure 4*B*. The interaction pattern of *FUS*_418_ is remarkably similar to that of *FUS*_390_, in which Arg and Lys predominate. Moreover, the number of interactions between Arg of RGG2 with RNA hairpin and Lys of RRM with RNA stem is higher than in other systems. In addition to these two residues, the Gly of RGG2 also exhibits several interactions specifically with the stem–loop junction of RNA. Notably, the interactions in *FUS*_418_ are uniformly distributed along the length of RNA, like Arg of RRM and RGG2 interacting with the RNA hairpin, Gly of RGG2 interacting with stem–loop junction, and Lys of RRM interacting with RNA stem, which maintains the structural integrity of RNA.

Since Arg and Lys residues are involved in both *FUS*_390_ and *FUS*_418_, Gly residues might have a crucial role in defining the RNA affinity of *FUS*_418_. The number and nature of these interactions might explain the augmented binding affinity of *FUS*_418_–RNA, and hence, the interactions are further classified as electrostatic, hydrophobic, and hydrogen bonds (Fig. S9). The residues Arg, Lys, Asp, and Phe are primarily responsible for electrostatic interactions, whereas the Gly residues are primarily responsible for hydrophobic interactions and a few hydrogen bonds. The major difference between *FUS*_390_ and *FUS*_418_ is the hydrophobic interactions between glycine and the stem–loop junction, stabilizing both the strands. Clearly, a large number of strong electrostatic interactions in *FUS*_390_, as compared with *FUS*_385_, may have a greater influence on the binding affinity as reported. However, the comparatively lesser increase in binding affinity between *FUS*_390_ (4.1 *μ*M) and *FUS*_418_ (2.5 *μ*M) is due to the addition of weak hydrophobic and H-bond interactions by the ∼28 Gly residues in RGG2. Though they are weak compared with the electrostatic interactions by Arg and Lys, they may collectively account for the increased RNA affinity of *FUS*_418_ over *FUS*_390_. Altogether, our study demonstrates that the number of RGG repeats has a direct effect on FUS–RNA interactions and affinity.

**Figure 5:**
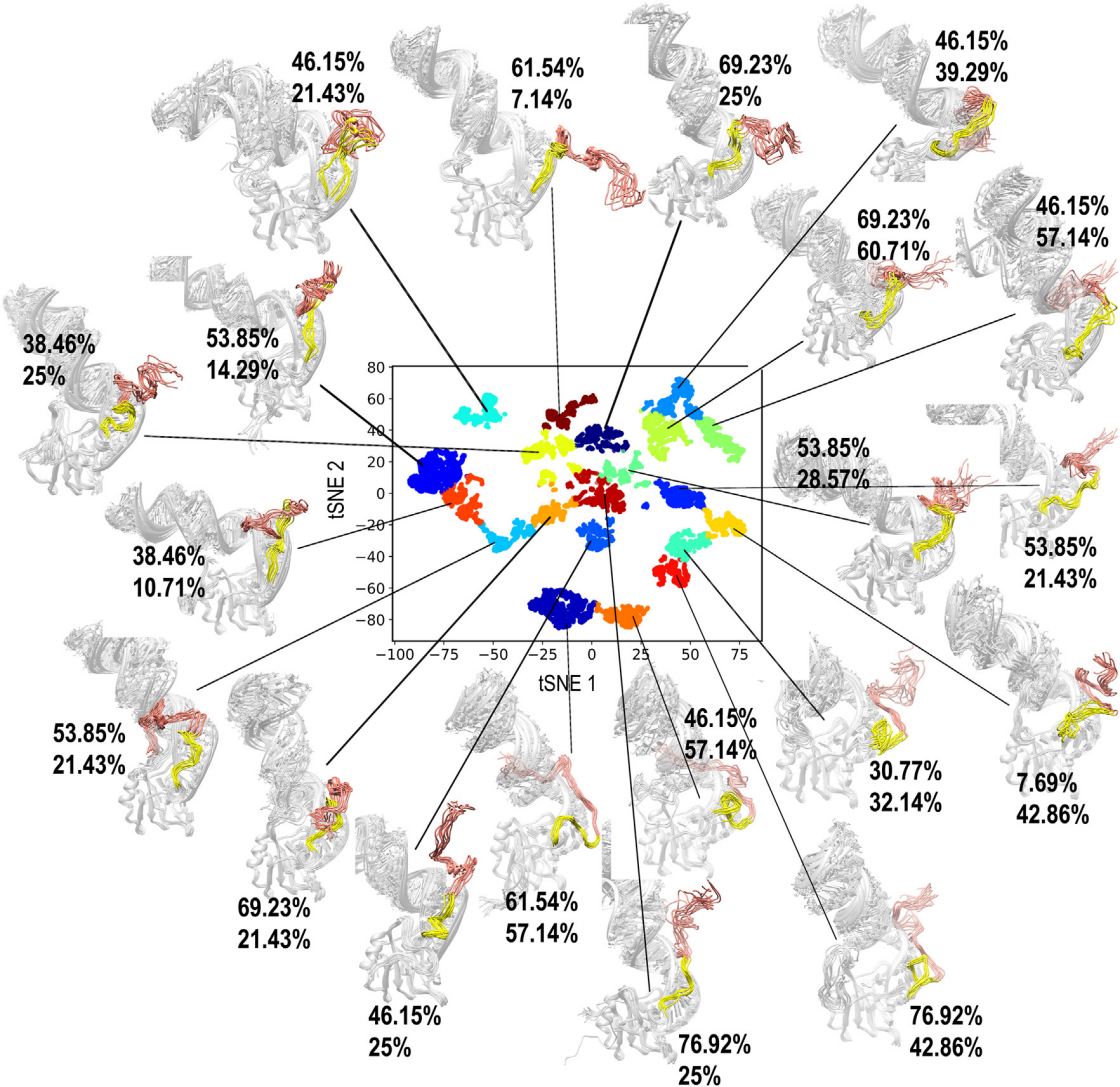
**Clustering of the *FUS***_**418**_**ensemble by t-SNE and K-means methods.** The projection of the first two t-SNE components classifies the sampled conformations into 20 distinct and unique clusters. Ten conformers from each cluster are superimposed, and the structures are mapped onto the projection. The stable RRM domain and RNA are shown in *gray*. The RGG2 can be further split into two independent regions (378–390 colored in *yellow* and 391–418 colored in *salmon*) based on their interaction pattern with RNA. The percentage of residues in these two regions that are in contact (*<*3.5 Å) with RNA is marked as percent in 378 to 390 followed by percent in 391 to 418. FUS, fused in sarcoma; RRM, RNA recognition motif; t-SNE, t-distributed stochastic neighbor embedding.

In addition to providing insights into the FUS–RNA interaction, our *FUS*_418_ simulation also allows us to explore the conformational landscape of an RNA-bound RGG. The heterogeneous conformations generated by our triplicate simulations were clustered from the 500 ns trajectory data using recently developed machine learning–based IDP-clustering method in our group. The method ingeniously combines the t-distributed stochastic neighbor embedding (t-SNE) projection data and kMeans techniques to cluster the highly heterogeneous IDP conformation ensembles into subgroups with homogeneous conformations (40). The algorithm is applied on our dataset to identify the distinct and unique conformations attained by the RGG2 when in complex with RNA. Figure 5 depicts the three-dimensional structures of 10 conformations extracted from each cluster. Each cluster is evidently highly homogeneous, whereas the conformations between clusters are heterogeneous. At least 52.31 ± 16.25% of the 13 residues in 378 to 390 AA remain in contact with the RNA throughout the simulation (percent of residues in contact with RNA in each cluster are shown in Fig. 5). In contrast, only 31.8 ± 15.9% of the 28 residues of 391 to 418 AA are in contact with the RNA. Among the individual clusters, the 378 to 390 AA exhibits a consistent interaction with RNA, whereas the number of residues of 391 to 418 AA that is in contact with RNA varies significantly between 7% and 60%. Altogether, these results demonstrate conclusively that RGG2 is essential for RNA binding and that it shows two distinct patterns for RNA binding: stronger binding with *<*390 AA and weaker binding with *>*390 AA.

### Flanking RGGs bind the entire RNA stem and further enhance RNA binding by FUS

**Figure 6:**
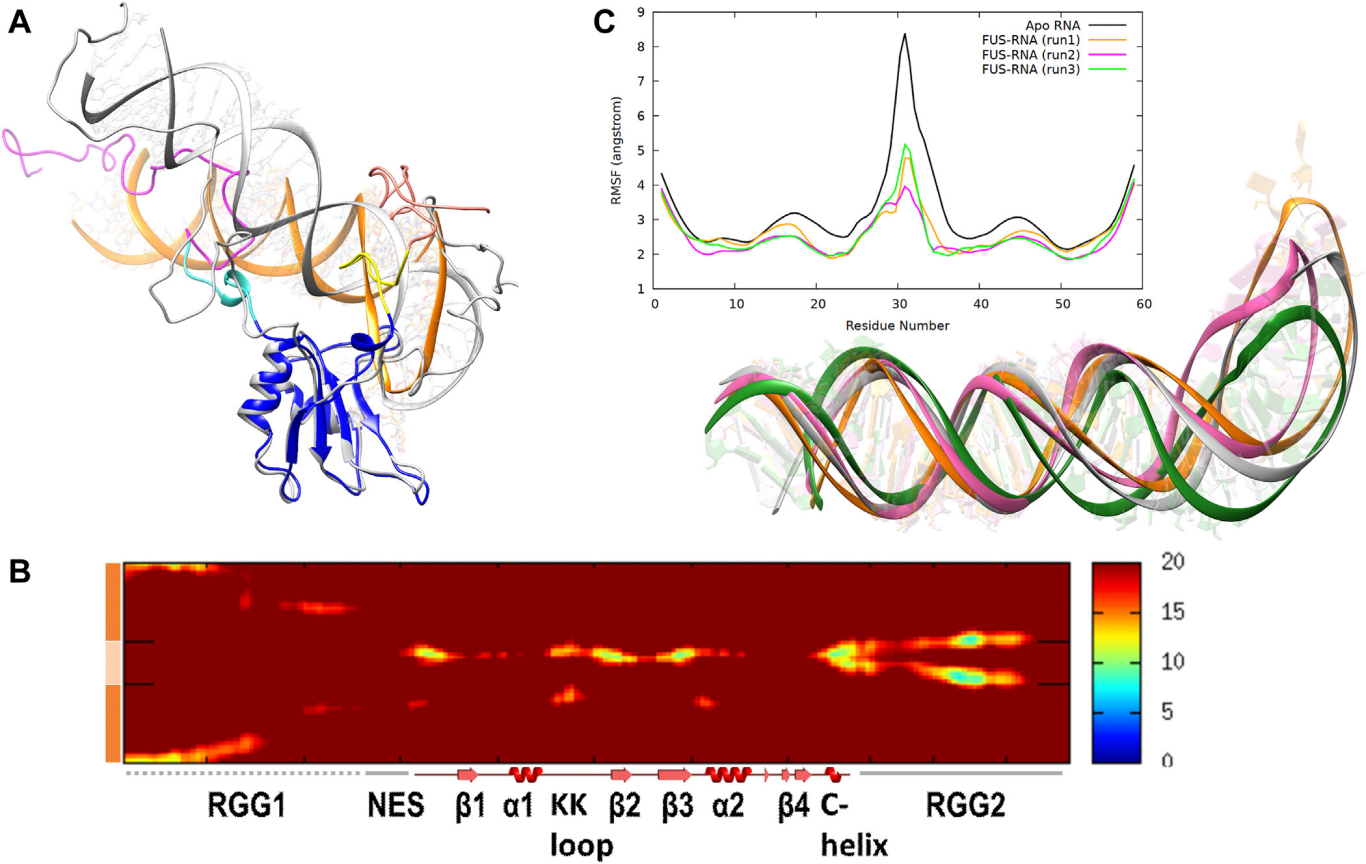
**Dynamics of *FUS***_**223−418**_**.***A*, superposition of the modeled FUS_223–418_–RNA complex in *gray* over the 1 μs simulated conformation of one of the three replicates. The different regions of FUS are colored as RGG1 in *magenta*, NES in *cyan*, RRM in *blue*, and the three simulated RNAs in *orange*, *pink*, and *green* colors, respectively. The RGG2 up to 390 (*yellow*) is colored distinctly from 390 to 418 (*salmon*) to highlight the importance of this region. *B*, the intermolecular distances (in Å) between the residues of FUS (223–418 AA) and RNA averaged over the last 100 ns of three replicate simulations. The secondary structures of FUS are represented on the *x*-axis, whereas the RNA stem (*dark orange*) and RNA loop (*light orange*) are represented on the *y*-axis. *C*, conformational dynamics of stem–loop junction RNA in complex with FUS (*orange*, *pink*, and *green*) and in solution (*gray*). The conformation of RNA simulated in solution (in *gray*) superposed with the conformation extracted after 500 ns simulation of the complex clearly highlights the stability of RNA. *Inset* shows the RMS fluctuation calculated from the three FUS–RNA complex simulations and one apo RNA simulation. (Detailed characterization of the RNA structural properties and the role of FUS in stabilizing its structure is an independent study.). FUS, fused in sarcoma; NES, nuclear export signal; RRM, RNA recognition motif.

The simulation of *FUS*_418_ clearly showed the distinct interaction pattern of RRM and RGG2 with the RNA hairpin and stem–loop junction, respectively. Even though the longer RGG2 could interact farther on the RNA stem in a fully extended state, our analysis has shown that the interactions are confined to the bases close to the stem–loop junction. The Arg residues in the RGG2 of both *FUS*_390_ and *FUS*_418_ show a very similar interaction pattern with the RNA hairpin and stem–loop junction, while the involvement of Gly in *FUS*_418_ is responsible for the slight increase in binding affinity. Interestingly, these Gly contacts are also limited to the stem–loop junction, whereas the farther stem regions remain free of any interactions. Since these interactions saturate at the stem–loop junction, intermolecular or intramolecular interactions from the other regions of FUS might further enhance the RNA-binding affinity. Accordingly, the presence of RGG1 (165–267 AA) with the RRM–RGG2 construct is reported to improve the RNA-binding affinity to ranges close to wildtype. Hence, in order to understand the role of RGG1 in RNA binding, we modeled the RG/RGG-rich part of RGG1 (223–267 AA), also in an extended conformation, similar to RGG2. Modeling an additional intrinsically disorded region (IDR) stretch of ∼50 AA to the RRM–RGG2–RNA complex is a nontrivial exercise. The RGG1 was added to a conformation of *FUS*_418_ chosen based on the number of RGG2–RNA contacts. There are five RG/RGG repeats in the 223 to 267 AA range, which might add several interaction sites for the RNA to bind efficiently. The modeled structure is shown in Figure 6*A*. The RGG1–RRM–RGG2 construct with 59 mer RNA, referred hereafter as *FUS*_223−418_, was simulated in triplicates of 1 μs each. The three-dimensional structure of the simulated complex is also shown in Figure 6*A* depicting the wrapping of RGG1 with the double-stranded RNA stem and RGG2 with the spine of the RNA-hairpin. Furthermore, the interatomic distances, as well as the residue-wise interactions, were also calculated to understand the FUS–RNA interactions.

**Figure 7:**
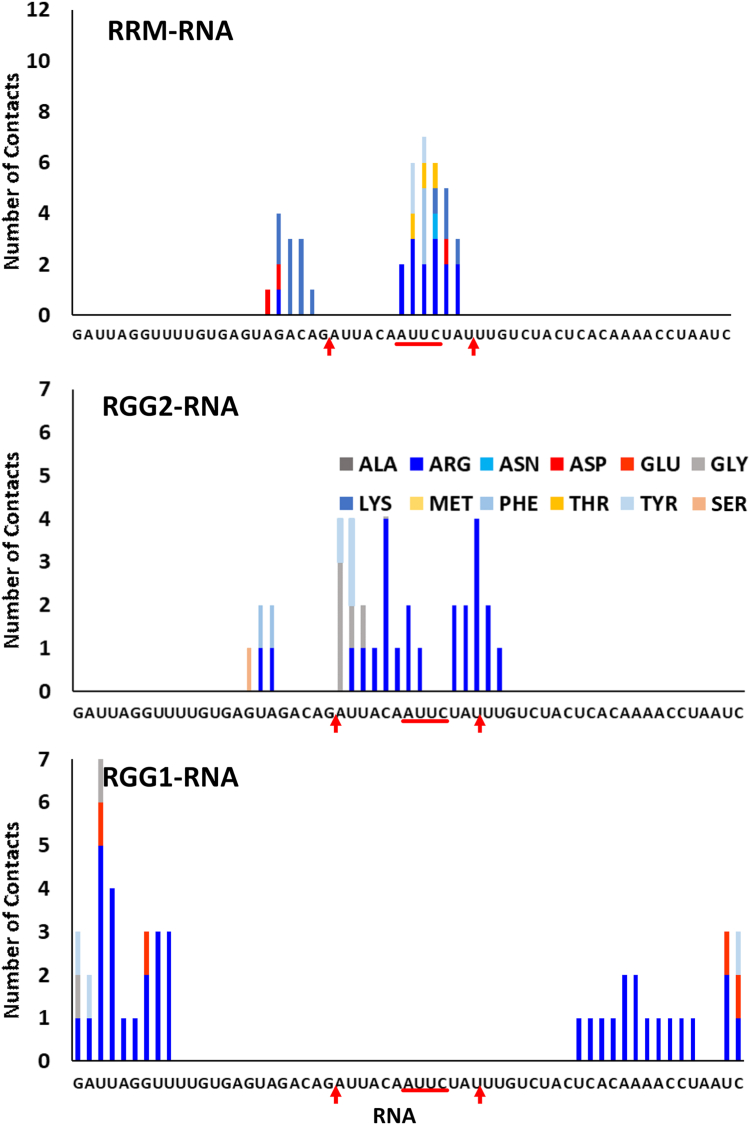
**Histogram depicting the number of interactions by each amino acid in the RRM, RGG2, and RGG1 domains with the individual bases of the 59mer RNA of FUS**_**223−418**_**.** The stem–loop junctions and the recognition motif are marked on the *x*-axis. FUS, fused in sarcoma; RRM, RNA recognition motif.

The interatomic distance map in Figure 6*B* clearly highlights the contacts formed by various regions of RGG1 and RGG2 with the entire length of RNA. The residues of RGG1 remain close to the RNA stem. In particular, the N terminus of RGG1 (230–250 AA) has high intensity with the ends of the RNA stem. RRM binds the RNA hairpin, whereas RGG2 is strongly in contact with the RNA stem–loop junction. In Figure 6*C*, we depict the RNA root mean square fluctuation along with a simulated APO RNA conformation and the conformation for the three replicas with RNA–protein complex. As compared with APO state, there is a noticeable drop in RNA root mean square fluctuation near the hairpin region when in complex with the protein. Moreover, the amino acid–wise interactions (shown in Fig. 7) highlight the division of labor by the various domains of FUS to stabilize the RNA by expressing strong electrostatic interactions. The Arg and Lys residues of RRM interact with the RNA hairpin, whereas the Arg residues of RGG2 interact with the stem–loop junction. However, interactions by Gly residues are lesser than *FUS*_418_, which is compensated by the stronger electrostatic interactions by Arg of RGG1 with both the strands of the RNA stem. In addition, Phe, Lys, and Asp also express a few interactions with the RNA stem. Interestingly, the interactions of RRM and RGG2 with the RNA are very similar to those seen in *FUS*_390_ and *FUS*_418_.

The three-dimensional structure of the simulated complex is also shown in Figure 6*A* depicting the wrapping of RGG1 with the double-stranded RNA stem and RGG2 with the spine of the RNA-hairpin. All the sampled conformations of *FUS*_223−418_ were clustered by t-SNE and K-means clustering to identify the distinct conformations, like *FUS*_418_. Because of the highly dynamic behavior of both RNA and RGG1, the conformational landscape is vastly heterogeneous, and hence, 70 distinct conformations are populated. In all the 70 clusters, shown in Fig. S10, the whole of RGG1 is seen to interact with the farther stem of RNA. Fig. S10 also clearly shows changes in the RNA conformation that might be in consequence to the FUS interaction. Overall, the addition of RGG repeats increases strong electrostatic interactions with RNA, and both the number (*FUS*_390_ *versus FUS*_418_/*FUS*_223−418_), as well as the position (RGG1 *versus* RGG2) of these RGG repeats, have a major influence on the binding of FUS with RNA.

FUS is a complex protein expressing an intricate network of interactions using the disorderedness of RGGs along with the properties of RRM-like KK loop and the RRM–RGG2 boundary. Since we have highlighted the crucial role of boundary residues in RRM–RNA complex throughout our study, we further tested this hypothesis in our larger FUS construct, FUS_223–418_. We identified three important residues 370-TRR-372 that interact with the RNA hairpin, based on our FUS_RRM_ and FUS_223-418_ simulations as well as the NMR structure of RRM (PDB ID: 6GBM). We further explored the importance of boundary region by modeling several mutant complexes of the three residues (T370A, R371A, and R372A and T370A/R371A/R372A mutant), and simulated the systems to see whether the mutants caused disruption in the complex stability. Within 100 ns of our simulations, we clearly see that the Ala mutations disrupt the interaction between boundary residues and RNA, thereby destabilizing the FUS–RNA complex. The instability of the mutant complex is depicted in Fig. S11, by monitoring the distribution of minimum distance between the β-sheet surface of RRM and the com of RNA hairpin to account for the RNA dynamics. This distance remains within 1.5 to 2 Å in the wildtype; however, it shifts rightward and fluctuates over a wider and larger distance range (1.7–3.0 Å) in the mutant systems implying the weakening of interactions between FUS and the RNA hairpin. Our mutant complexes further reinforce the importance of boundary region in RNA binding and affinity.

### FUS-RRM requires RNA sequence/shape specificity to initiate RNA binding

The RRM domain of FUS is reported to express shape specificity, and accordingly, Loughlin *et al*. (23) proposed a consensus sequence motif of NYNY or YNY (Y = C/U; N = A/G/C/U) for the recognition. The hnRNP A2/B1 pre-mRNA sequence used in our study comprises of AUUC motif at the recognition site, and as we saw in the previous sections, this motif interacts well with the RRM domain. For the recognition to happen, the “Y” position in the NYNY motif should contain an “O2” atom as in cytosine or uracil. By mutating this position to adenine or guanine, we posited that the specificity should be lost, and therefore, the RRM–RNA interaction should be weaker. In order to test the presence of any sequence or shape specificity in RNA recognition by RRM, we mutated the AUUC motif into AAUG in the NMR structure of *FUS*_390_ (since experimental structure is known) and the simulated conformation of *FUS*_418_.

**Figure 8:**
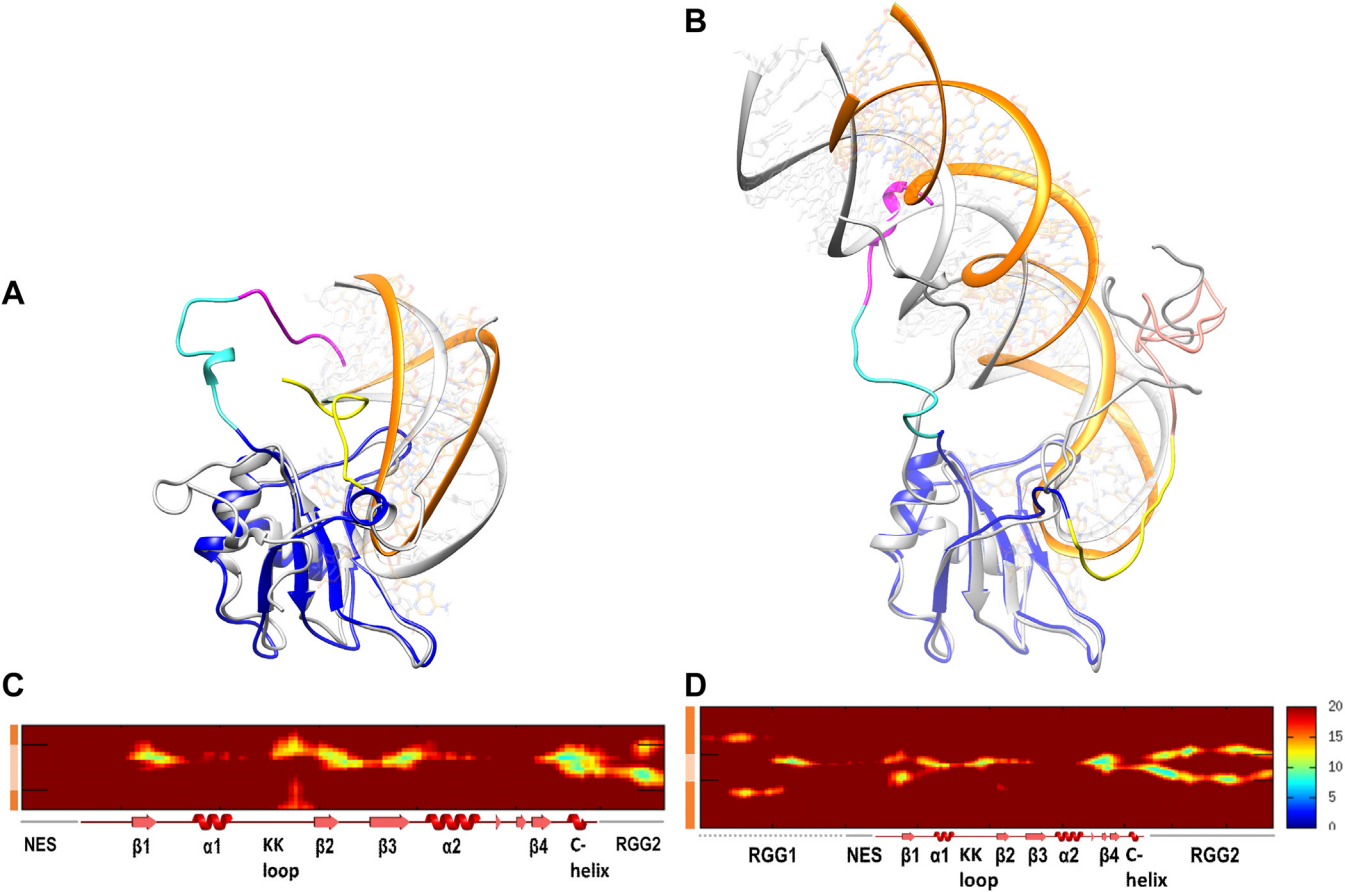
**Dynamics of FUS in complex with RNA mutant**. Structure superposition of initial (*gray*) and 1 μs simulated conformations of (*A*) *FUS*_390_−*RNA*_*mut*_ and (*B*) *FUS*_418_−*RNA*_*mut*_. The different regions of FUS in the simulated conformations are colored as RGG1 in *magenta*, NES in *cyan*, RRM in *blue*, and RNA in *orange*. The RGG2 up to 390 (*yellow*) is colored distinctly from 390 to 418 (*salmon*) to highlight the importance of this region. The intermolecular distances (in Å) between FUS and RNA in (*C*) *FUS*_390_−*RNA*_*mut*_, (*D*) *FUS*_418_−*RNA*_*mut*_ averaged over the last 100 ns simulation. The secondary structures of FUS are represented on the *x*-axis, whereas the RNA stem (*dark orange*) and RNA loop (*light orange*) are represented on the *y*-axis. FUS, fused in sarcoma; NES, nuclear export signal; RRM, RNA recognition motif.

The *FUS*_390_−*RNA*_*mut*_ and *FUS*_418_−*RNA*_*mut*_ complexes were modeled and simulated for a period of 1 μs, and the superimposition of initial and 1 μs simulated conformations is shown in Figure 8, *A* and *B*. The three-dimensional structure clearly shows that the binding of mutant RNA in *FUS*_390_−*RNA*_*mut*_ is highly disrupted in contrast to the wildtype RNA in *FUS*_390_. Also, the single turn of the C-terminal helix in wildtype RNA complex is extended to include another turn leading to the reorientation of the RGG2 away from the RNA. These major conformational changes were not observed in any of the triplicate trajectories of wildtype RNA complex in *FUS*_390_ suggesting a weak interaction of mutant RNA with RGG2. The interatomic distance matrix calculated over the last 100 ns of mutant RNA complex simulations is shown in Figure 8, *C* and *D*. The *FUS*_390_−*RNA*_*mut*_ shows slightly reduced intensities for RRM–RNA and much weaker intensities for RGG2–RNA indicating unstable RNA binding. Contrastingly, in the case of *FUS*_418_−*RNA*_*mut*_, the interatomic distance for RRM–RNA is very similar to wildtype RNA complex. Though the 378 to 390 AA remains close to RNA, the G-rich part of RGG2 (391–418 AA) reorients closer to the RNA hairpin. This is also shown in Figure 8*D* where the intensities are entirely absent for the G-rich part of RGG2.

**Figure 9:**
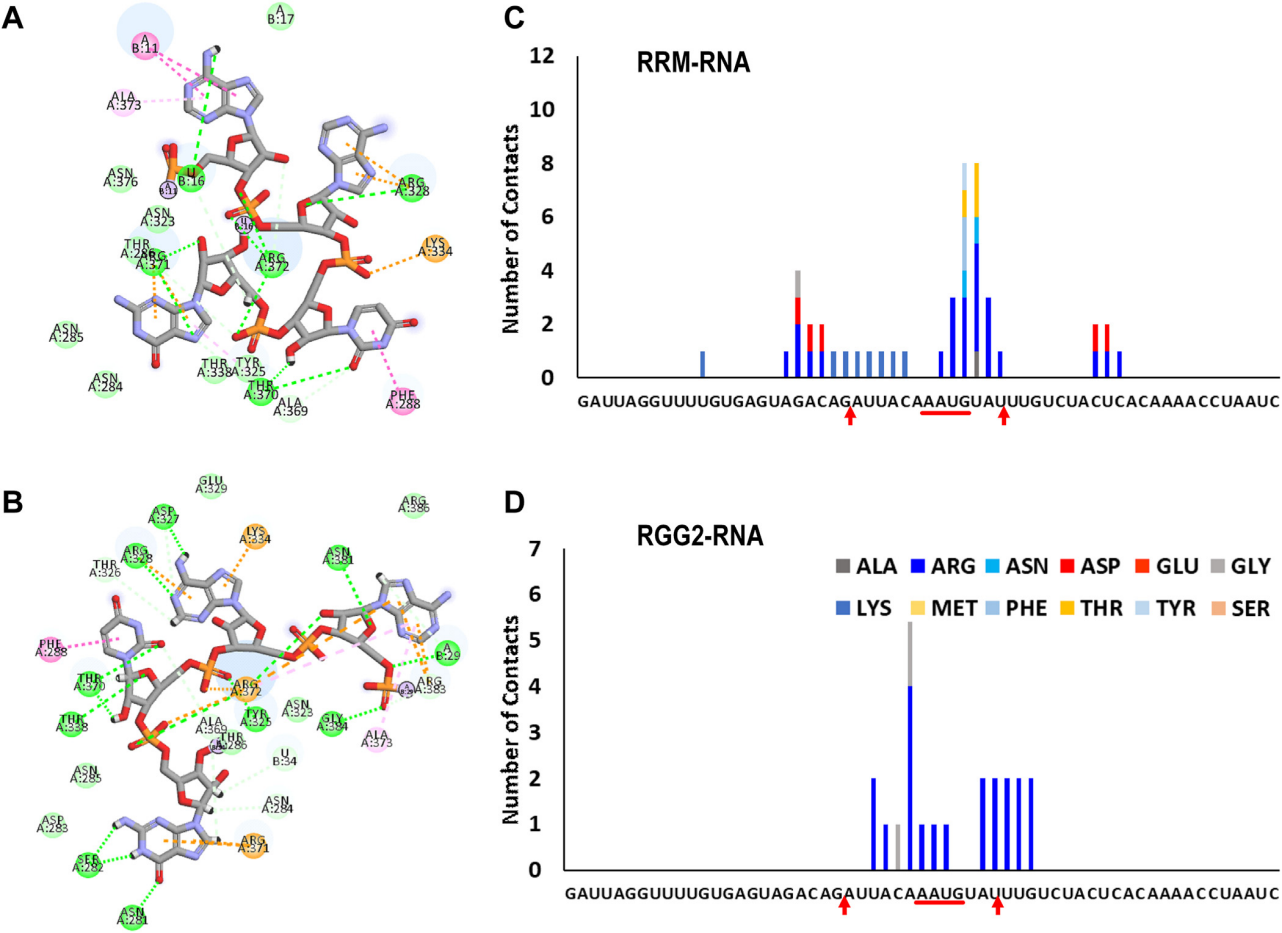
**Interactions in FUS-RNAmut complexes.** The two-dimensional interaction diagram depicting the different residues interacting with the AUUC(AAUG) motif of RNA in (*A*) *FUS*_390_−*RNA*_*mut*_ and (*B*) *FUS*_418_−*RNA*_*mut*_. *Green dotted lines*: hydrogen bonds, *orange dotted lines*: *π*–cation interactions, *pink dotted lines*: *π*-stacking interactions, *pale green discs*: hydrophobic interactions. Amino acid–wise interactions depicting the number of interactions by each amino acid in the (*C*) RRM and (*D*) RGG2 domains with the individual bases of the 59mer RNA in *FUS*_418_−*RNA*_*mut*_. FUS, fused in sarcoma; RRM, RNA recognition motif.

The mutated AAUG motif in *FUS*_390_−*RNA*_*mut*_ and *FUS*_418_−*RNA*_*mut*_ shows few conserved and several new interactions with the RRM domain (Table S1 and Fig. 9, *A* and *B*). The mutation of U in the second position to A allows several additional interactions to form in both the mutant complexes, whereas none of the interactions from *FUS*_*RRM*_ or NMR are conserved for C to G in the fourth position. The stacking interaction of U in the third position with Phe288 is still conserved along with hydrogen bonds with the backbone of Thr370 and Arg372. Apart from this, there are several new interactions with the *β*2–*β*3 loop (residues Asn323, Tyr325, and Arg328), C-terminal helix (Thr370, Arg372, and Ala373), and Arg386 of RGG2. When compared with the wildtype complexes, it is clear that the RNA orientation in both the mutant complexes is different and the KK loop is devoid of any interactions. Our results from previous sections have highlighted the importance of hydrophobic interactions by the Gly residues of the extended RGG2 (391–418 AA) to stabilize RNA. Hence, these interactions were further analyzed in *FUS*_418_−*RNA*_*mut*_ to explore the importance of this extended RGG2 on RNA binding. The interaction histogram of mutant RNA with RRM and RGG2 of *FUS*_418_−*RNA*_*mut*_ system detailing the contribution of each amino acid to RNA binding is shown in Figure 9, *C* and *D*.

It is interesting to note that the mutation in the RNA motif recognized by the RRM domain clearly affects the binding and pattern of the remote interactions in the RGG2 loop, particularly with the G-rich half of RGG2 (391–418 AA) and its Gly residues. First, the Gly residues are not dominating the interactions. Several new interactions between Arg of RRM domain and the RNA stem–loop junction as well as the RNA stem are seen, which is very unique to the mutant RNA complex. Similarly, another unique interaction is seen between the RNA hairpin and Lys residues. It is worth mentioning here that the *β*-sheet surfaces, where the RNA hairpin is supposed to interact, are entirely devoid of Lys residues apart from the KK loop and *β*2–*β*3 loop. Even though *FUS*_*RRM*_ was unable to stably bind the entire RNA, the recognition motif was interacting strongly. However, in the case of AAUG RNA mutant, the recognition motif loses several interactions with the *β*-sheets of RRM. The loss of these interactions with the RRM is clearly seen to be compensated by stronger electrostatic interactions with the Arg/Lys of both RRM and RGG2. Hence, it is clear that the interaction of RRM with the recognition motif in the mutant RNA is severely disrupted, nevertheless, parts of RGG2 were able to hold the RNA stem to still remain interacting with FUS.

These observations also highlight the importance of RGG2 for RNA binding and the drastic enhancement of binding affinity because of the inclusion of a minimal number of RGG repeats. Collectively, it can be hypothesized that the specificity of RRM to RNA sequence/shape is required only for the initial recognition or localization, and thereafter, the interactions with RGG are stronger to overcome any loss in sequence/shape specificity. This highlights the synergistic effect and division of labor among the various regions of FUS protein, where the loss of interaction with one of the domains might be compensated by the gain of interactions with the other domains of FUS.

### Force fields and sampling considerations for simulations of IDP–RNA complexes

Force fields play a crucial role in the simulation of biomolecules. The governing principles of the structure of a folded and disordered protein are vastly different requiring additional parameters to model the intrinsic disorderness. Further adding to this challenge is the representation of IDPs and multidomain proteins with a mixture of IDRs and folded domains. Recent developments in all-atom force fields have greatly improved the modeling of IDPs by modifying the protein-solvent behavior. a99SB-disp is one such forcefield with the ability to model proteins with both ordered and disordered domains (41). The performance of IDP-compatible versions of Amber (42), CHARMM (43) and D.E. Shaw (41, 44) forcefields was explored previously by Sarthak *et al.* (33) using FUS as the model system. Since amber ff14 and a99SB-disp were used by Pokorná *et al.* (32) and Sarthak *et al.*, respectively, to study a very similar FUS–RNA complex, we tested the performance of both these force fields for FUS alongside OL3 (45) and DES-amber (46) for RNA. A detailed discussion of these test systems is included in the Supporting information. In Fig. S12, we show the effect of three different force fields on *FUS*_*390*_–RNA complex. Our test systems clearly highlighted the significant effect of force fields on FUS dynamics reinforcing the importance of choosing a force field compatible to the system of interest.

Another major issue in MD simulations of IDPs is the sampling of their conformational landscape. Being a protein without a defined three-dimensional structure, the conformational landscape of IDPs is very vast requiring a larger sampling. Previously, the structure and dynamics of IDPs like Tau proteins and LAF-1 (47, 48, 49) were investigated by classical all-atom MD simulations. Enhanced simulation methods like replica exchange and metadynamics are useful for the sole purpose of exploring the structure landscape since classical MD tends to sample the conformations close to local minima. In this work, we have carried out canonical MD simulations. To generate a significantly large ensemble of conformations, we performed simulations of each system with three replicates of 1 μs each. We do acknowledge that had we done a much longer simulation (*e.g.*, the 10 μs simulations carried out by Sarthak *et al.* on FUS-RRM system), better statistics and convergence as well as information on association/dissociation kinetics could be more faithfully achieved. Since the main purpose of our study is to explore the interaction pattern of an existing FUS–RNA complex, we believe (based on our convergence studies) that a sampling of 1 μs and three qualitatively similar behaving trajectories provides substantial information regarding the FUS–RNA interaction mechanisms.

## Conclusion

In this article, we have used large-scale (>21 μs total run) molecular simulations at an all-atom resolution to understand the atomic-level interactions in FUS–RNA complexes. Our study provides molecular-level mechanistic insights into observations from biochemical studies, and it has also illuminated our understanding of molecular driving forces that mediate the structure, stability, and interaction of RRM and RGG domains of FUS with a stem–loop junction RNA. Our simulation data clearly bring forth the very important role of the boundary region at the interface of RRM and RGG2, which seems to be central to the fidelity of the complex. This region is ambiguously classified as either RRM or RGG2 causing inconsistency in comparing the binding affinities among various experimental literature. We show that excluding this region in RRM or their mutations lead to dissociation of RNA, and this is an experimentally testable hypothesis. With FUS-RRM devoid of the classical recognition motifs seen in other RRMs, we believe that this boundary region between the folded and disordered domains gains importance as the anchor along with the earlier discovered noncanonical central KK loop. Our study also provides the structural biophysical rationale for why at least three RGG repeats are required in RGG2 to improve binding to the RNA. We find that the Arg residues in the first two RGG repeats are sterically constrained because of the persistence caused by the small helix at the start of RGG2. We also find that whatever the length of RGG2 is, the interactions are confined to the RNA hairpin and stem–loop junction only. The fourth and fifth RGG repeats in RGG2 do not significantly improve the binding strength. However, based on our data, the mild increase that was observed in experimental studies could be attributed to the hydrophobic interactions between glycines and RNA. The role of glycine residues in biomolecular interactions has been widely overlooked, yet we have observed an important role for these interactions in increasing the affinity of FUS–RNA complex. This is interesting from the point of view of bounds put on RGG repeats that maximizes their functional role. On the other hand, we find that once RGG1 is introduced from the N-terminal end of the RRM, RNA binding noticeably increases again. Flanking RGGs bind the entire RNA stem, and our simulations provide a very clear picture of the origins of the enhanced interactions. Interestingly, the nuclear export signal region connecting RGG1 with RRM does not express any interactions with the RNA, thereby facilitating the recruitment of other binding partners. Our data from RNA mutation simulations again provide experimentally testable hypotheses to establish the RNA sequence and structure specificity of FUS protein where we see specificity for NYNY motif. Mutation directly alters the RRM–NYNY interaction pattern, and as a result, we observe an indirect allosteric effect in the RGG2. Such adaptable interactions of FUS are mainly responsible for its promiscuous nucleic acid–binding property and minimal sequence specificity. RGGs are considered as the key component in an interaction network because of their ability to express intramolecular as well intermolecular interactions with other RGGs, disordered or folded proteins, and nucleic acids. Because of such high multivalency, RGG domains also contribute significantly to liquid–liquid phase separation and aggregation of proteins like FUS. Previous studies have highlighted weak interaction of RGG with the LCD of FUS and also with the RNA polymerase II in a condensate *via* Arg or Lys of RGG and Tyr of LCD. Similarly, our study identified the interaction of FUS RGG with RNA mediated by Arg, Lys, and Gly residues. Together, the Arg and Lys residues of RGG are crucial for the function of FUS since they interact with both LCD and RNA, thereby also facilitating RNA concentration–dependent phase separation of FUS. Further exploring the RGG–LCD and RGG–RNA interactions in the context of full-length FUS would greatly help in understanding the liquid–liquid phase separation of FUS.

## Experimental procedures

### MD simulations

MD simulations of FUS–RNA complexes were carried out using the GROMACS package. Table 1 lists the FUS–RNA complex systems under consideration. Combining the findings of Pokorná *et al.* (32) and Sarthak *et al*. (33), our study utilizes the a99SB-disp force field (41) to model FUS and OL3 force field (45) to describe the RNA. We also tested combinations of other force fields like ff14SB for protein and DES Amber for RNA; however, these force fields increase rigidity in the system. The choice of force field, their limitations, and rationale are discussed in supporting information. In recent years, the forcefield a99SB-disp has been used successfully to sample proteins containing both folded and disordered regions, and hence, this force field was used in our study (33). These complexes were solvated with a99SB-disp-specific TIP4P-D water in a periodic box with an additional water pad extending up to 12 Å in all directions. The systems were neutralized, and sufficient ions were added to mimic 150 mM salt concentration. Table S2 provides information regarding the number of atoms in each simulated system, the dimensions of each simulation box, and the number of ions added to each system. Also, a figure depicting the simulation box with FUS_223–418_–RNA complex, ions, and water is shown in Fig. S13. The short-range interactions were truncated with a cutoff distance of 10 Å. Electrostatic interactions were treated by particle-mesh Ewald with a real space cutoff value of 10 Å. Bonds containing hydrogens were constrained using the LINCS algorithm. The solvated and neutralized systems were energy minimized using the Steepest Descent algorithm, followed by a 5 ns equilibration, and subsequently, production runs were taken up. The temperature and pressure of the systems were maintained at 310 K and 1 Atm using the Nose–Hoover thermostat and Parrinello–Rahman barostat in an NPT ensemble. The simulations were performed in triplicate of 1 μs each to improve sampling and the significance of our results. All analyses were performed over the trajectories saved during the production runs, with the GROMACS analysis tools and CPPTRAJ module of AmberTools20. Xmgrace, UCSF Chimera version 1.13, and VMD 1.9.3 were used for visualization and preparing the images.

### Modeling RNA stem–loop structure

Our study utilized the NMR structure of a stem–loop RNA formed by the hnRNP A2/B1 pre-mRNA sequence in complex with the FUS-RRM domain (PDB ID: 6GBM (23)). Other FUS–RNA complexes were modeled using this 23mer stem–loop RNA by superposing the RRM domains. The hnRNP A2/B1 pre-mRNA sequence containing the bipartite motif (RRM-specific AUUC and ZnF-specific GGU) was used to extend the length of this RNA. This sequence, used by Loughlin *et al*. (23), consists of an RNA hairpin and a single-stranded stem. The extended RNA structure was modeled as a double strand by extending the complementary strand also in order to use a stable RNA structure while modeling the flexible RGG loops. The RNA structure was modeled using Discovery studio visualizer 2019, and the *FUS*_418_ complex structures were modeled based on the binding orientation of the 23mer RNA hairpin in 6GBM by superimposing the RRM domains. The 23mer RNA hairpin has the following sequence: GGCAGAUUACAAUUCUAUUUGCC. The following sequence was used by Loughlin *et al.* (23) for the bipartite motif [GAUUAGGUUUUGUGAGUAGACAGAUUACAAUUCUAUUUUAA], and we use an extended sequence as described previously and given as:

[GAUUAGGUUUUGUGAGUAGACAGAUUACAAUUCUAUUUGUCUACUCACAAAACCUAAUC]

### Modeling of RGG1 and RGG2 stretches

Computational modeling of IDP and IDR structures (50, 51) is a challenging process because of their heterogeneous conformations landscape. Also, IDRs that follow the “folding upon binding” principle generally require their interaction partners to attain a properly folded state. There are several integrative modeling and pure simulation methods, both at all-atom resolutions and reduced resolutions, which can be used to elucidate the conformational ensemble of IDP–IDRs in their APO state (41, 52, 53, 54, 55, 56, 57, 58, 59, 60, 61, 62, 63, 64). In our study, in which the IDR must be modeled in complex with the RNA, we add the IDR in fragments and have modeled the RGG repeats undergoing the “fuzzy interaction” mechanism using classical all-atom MD simulation of the interacting partners. The sequences of RGG2 (391–418 AA) and RGG1 (223–269 AA) were divided into fragments of 3 to 5 AA (eight fragments for RGG2 and seven fragments for RGG1), and each fragment was added successively. During this process, the FUS domains, including the RNA (RRM–RNA for RGG2 modeling and RRM–RGG2–RNA for RGG1 modeling), were restrained to their initial position with a harmonic restraint weight of 10,000 kJ. The restraints on RNA and parts of FUS would ensure that the newly modeled regions express unrestricted dynamics in order to achieve a well-bound state. Each fragment was added to the restrained FUS–RNA complex and simulated for a period of 50 ns. After the 50 ns restrained simulation, the trajectories were visually examined for their interaction with RNA to ensure that the RGG is bound to the RNA. In this bound conformation, the residues at the C (for RGG2) or N (for RGG1) terminus were in an extended state, permitting further extension of RGGs by adding another fragment using the same protocol. Fig. S14 depicts the fragments and intermediate structures during this modeling protocol, highlighting the addition of RGGs in fragments. The RGG regions were added sequentially to the RRM (PDB ID: 6SNJ (65)). In other words, the RGG2 was added to the RRM-RNA construct (*FUS*_*390*_) first, followed by the modeling of the RGG1 into the system. After modeling each RGG, all harmonic restraints were removed, and the structures were simulated using the standard MD simulation protocol as explained previously.

### Interaction analysis

We use three different distance-based metrics in this study, namely, the com distance, minimum distance, and interatomic distance matrices. The com distance was calculated between the com of RRM and com of RNA as a whole. This distance would be in the range of 20 to 25 Å and indicates the overall binding orientation of RNA with respect to the RRM. The second metric, namely the minimum distance, was calculated between the beta-sheet surface of RRM (286–290, 322–324, and 336–340 AA) and the RNA hairpin (bases 6–18 in the 23mer RNA and the equivalent 24–36 in the 59mer RNA). The minimum distance between any pair of atoms from these two groups is calculated, and this distance signifies the closest range that these two groups can achieve. By monitoring the changes in com–com distance and minimum distance, we can identify the RNA dynamics. Changes in minimum distance indicates dissociation of RNA. However, a stable minimum distance can also be accompanied with fluctuations in com–com distance, signifying disruptions in the binding orientation of RNA relative to RRM. The third distance metric, namely the interatomic distance matrix, is a matrix of distances between all the atoms of RNA and all the atoms of FUS averaged over 100 ns simulation. The matrix is plotted as a two-dimensional landscape with FUS residues on the *x*-axis and RNA on the *y*-axis. We have used a blue to red scale to color the residue pairs that are closest to farthest. We have used a cutoff of 20 Å to color the distances; hence, all residue pairs beyond 20 Å are shown in red. This distance ensures that all interacting residues and interaction types including electrostatic, *π*-, hydrogen bonds, and hydrophobic interactions are accounted for during the calculation. This matrix is helpful to identify the regions of FUS interacting with particular regions of RNA and the changes in binding orientation between the different simulated systems. The amino acid–wise interaction plots were calculated as an average of the last 100 ns of the three independent simulations. For the histogram of interacting residues, Cpptraj module of AmberTools20 was used to extract all pairs of residues between FUS and RNA present within a 6 Å distance that is maintained for at least 10% of the simulation period. The interactions by each RNA base were grouped on the interacting amino acids and the number of these interactions is plotted. Since all residue pairs within 6 Å are considered, the obtained number includes all types of nonbonded interactions like hydrogen bonds, electrostatic, *π*-, and hydrophobic interactions. Furthermore, these interactions were classified based on similar studies done previously (66).

### Uniform clustering of simulated IDR-RNA ensemble using t-SNE

MD simulation generates an ensemble of conformations representing the dynamics of biomolecules, and valuable insights could be derived by clustering these conformations. The clustering of an IDP ensemble is a challenging task because of the high conformational heterogeneity. Several clustering methods like hierarchal, vector quantization, and neural network are available to perform the clustering analysis. In our study, we use the nonlinear dimensionality reduction method called t-SNE coupled with the K-means method for clustering the highly heterogeneous IDP/IDR ensemble of FUS into subgroups of homogeneous conformations. Complete details about this method for clustering IDPs are available in the recent article from our group (40). The clustering was driven by calculating the RMSD of every conformation with every other conformation, extracted at 50 ps interval, to represent the similarity/dissimilarity among the ensemble. The RMSD was calculated for the RGG2 region while superposing the stable RRM domain in order to account for the dynamics of RGG2 alone. The major advantage of t-SNE algorithm is the tunable parameter called perplexity value, which can balance the information between the local and global features of our dataset. The choice of perplexity value is important for dividing the data into discrete and unambiguous clusters. In this work, different perplexity values and the number of K-means clusters were explored, and the combination that gave us the best possible Silhouette score was used to undertake the clustering exercise. Our in-house code and SciKit, an open-source library for Python-based machine learning, were used to perform these analyses and publicly available in github repository.

## Data availability

Input files needed to initiate molecular simulations and full trajectory data of all simulations for all systems considered in this work are available on our server for download. The server data can be publicly accessed *via* our laboratory GitHub link: codesrivastavalab/RNA-FUSAAMD. The files can also be accessed directly from the publicly available Figshare portal: https://doi.org/10.6084/m9.figshare.24155325.v1.

## Supporting information

This article contains supporting information (32, 33, 42, 43, 44, 46, 67).

## Conflict of interest

The authors declare that they have no conflicts of interest with the contents of this article.
