# Supplementary material for: “Boundary residues” between the folded RNA recognition motif and disordered RGG domains are critical for FUS–RNA binding
Source: J Biol Chem. 2023 Oct 27;299(12):105392. doi: 10.1016/j.jbc.2023.105392 (PMC10687056; doi:10.1016/j.jbc.2023.105392)
Supplement: Supporting information [file mmc1.pdf]

Supporting Information

"Boundary residues" between the folded RNA Recognition Motif and disordered RGG domains are critical for FUS-RNA binding

Sangeetha Balasubramanian,[1] Shovamayee Maharana,[2] and Anand Srivastava[1, *1]

[1)] *Molecular Biophysics Unit, Indian Institute of Science Bangalore, C. V. Raman Road, Bangalore, Karnataka 560012, India*

[2)] *Department of Molecular and Cell Biology, Indian Institute of Science Bangalore, C. V. Raman Road, Bangalore, Karnataka 560012, India*

Fig. S1-S11: Figures supporting the dynamics of FUS-RNA complexes

Fig. S12: Force field Limitations

Fig. S13: Depiction of the simulation box

Fig. S14: Depiction of the RGGs modeling protocol

Table S1: List of FUS-RNA interactions and their lifetimes

Table S2: The description of the FUS systems simulated in this study

*1 Electronic mail: anand@iisc.ac.in

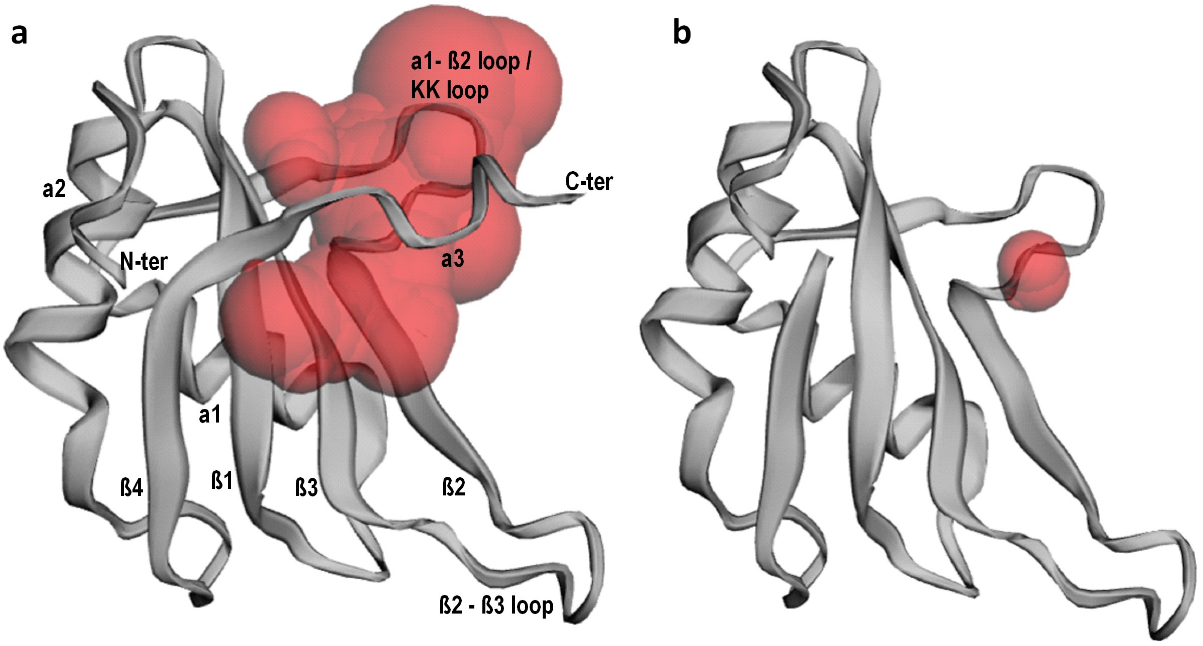

Fig. S1: Binding pocket volume analysis using the CASTp server for (a) FUS-RRM (276-377 aa) and (b) truncated FUS-RRM (276-368 aa) to depict the role of the C-terminal helical turn in increasing the volume of RNA binding pocket (red surface representation).

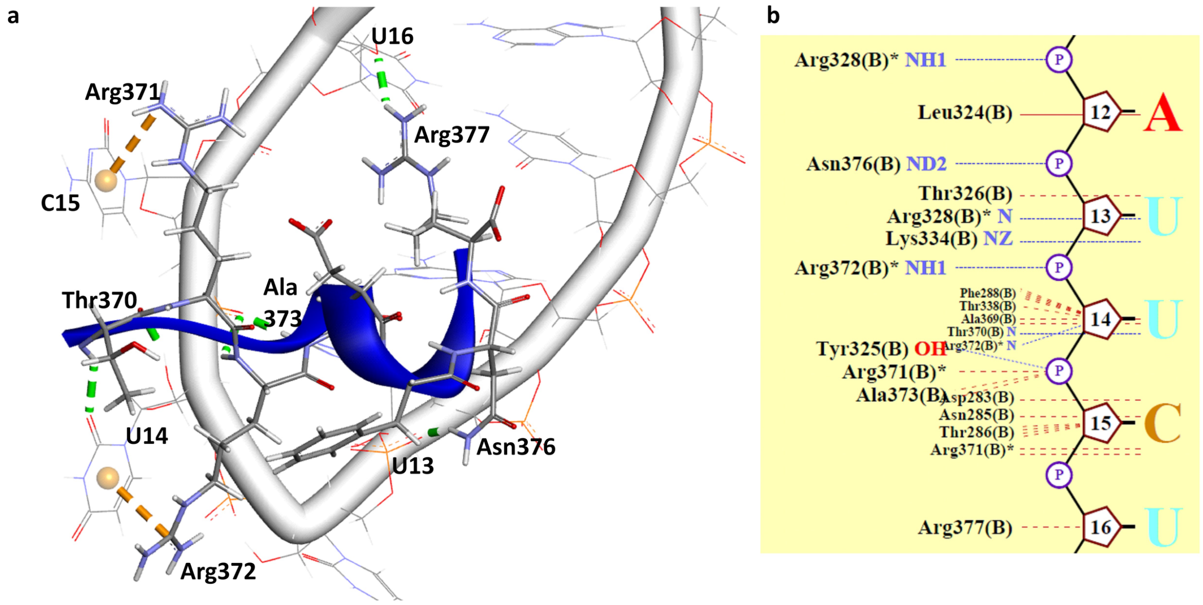

Fig. S2: Interaction of C-terminal boundary region (369-377 AA) with RRM in the NMR structure with PDB ID: 6GBM in (a) three-dimensional and (b) two-dimensional representations. FUS is shown in Blue cartoon with the sidechains depicted in Licorice, colored based on the atoms. The RNA backbone is shown as a tube with the bases displayed as wires. The Hydrogen bonds are shown as Green dotted lines, while the π-interactions are shown as Orange dotted lines. The FUS residues and RNA bases involved in these interactions are labeled. In the two-dimensional representation, generated using PDBSum [67], Blue dotted lines indicate Hydrogen bond and Red dashed lines indicate all nonbonded contacts within 3.35Å.

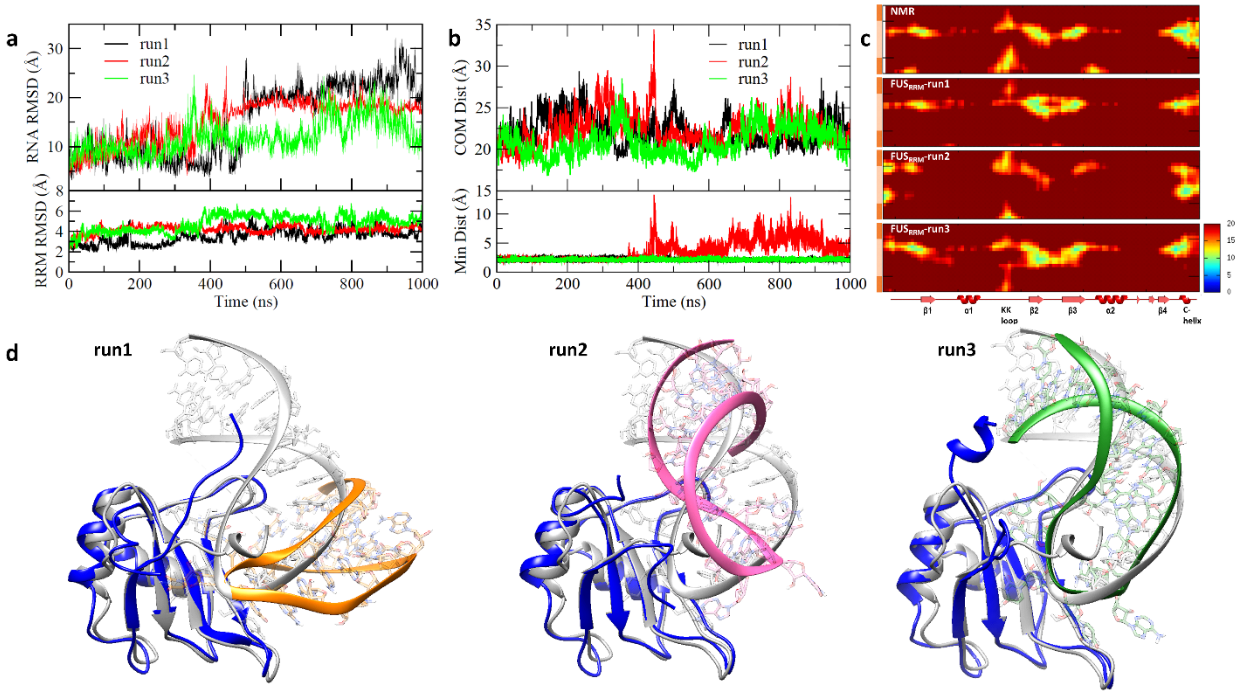

Fig. S3: Dynamics of FUS_{RRM}. (a) Time evolution of all-atom RMSD of FUS_{RRM} (bottom panel) and RMSD of RNA (top panel) calculated with respect to the RRM domain as reference defining the stability of the RNA binding orientation. All three replicates show stable RRM while the binding orientation of RNA is highly dynamic. (b) Variation in the center of mass distance (top panel) as well as the minimum atom-pair distance (bottom panel) between the RRM surface and RNA hairpin. In accordance to the RMSD variation, the com-com distance of RRM and RNA varies considerably, however, the minimum atom-pair distance shows that the RNA hairpin remains in contact with the RRM throughout the 3 µs simulation. (c) The inter-atomic distances between the residues of FUS_{RRM} (276-377 aa) and RNA averaged over the last 100 ns simulation. The secondary structures of FUS are represented on the x-axis, while the RNA stem (dark orange) and RNA loop (light orange) are represented on the y-axis. The binding mode of RNA when compared to the NMR structure is inconsistent, however, the RNA hairpin remains in stable contact with the β-sheet surfaces and the RNA stem dynamics alone varies. (d) Structure superposition of initial (gray) and 1 µs simulated conformations of FUS_{RRM}. The different regions of FUS in the simulated conformations are colored as NES in cyan, RRM in blue, and the three RNAs are colored in orange, pink and green. The binding orientations of RNA varies considerably in the three independent simulations, yet, we do not observe total dissociation of RNA from the RRM.

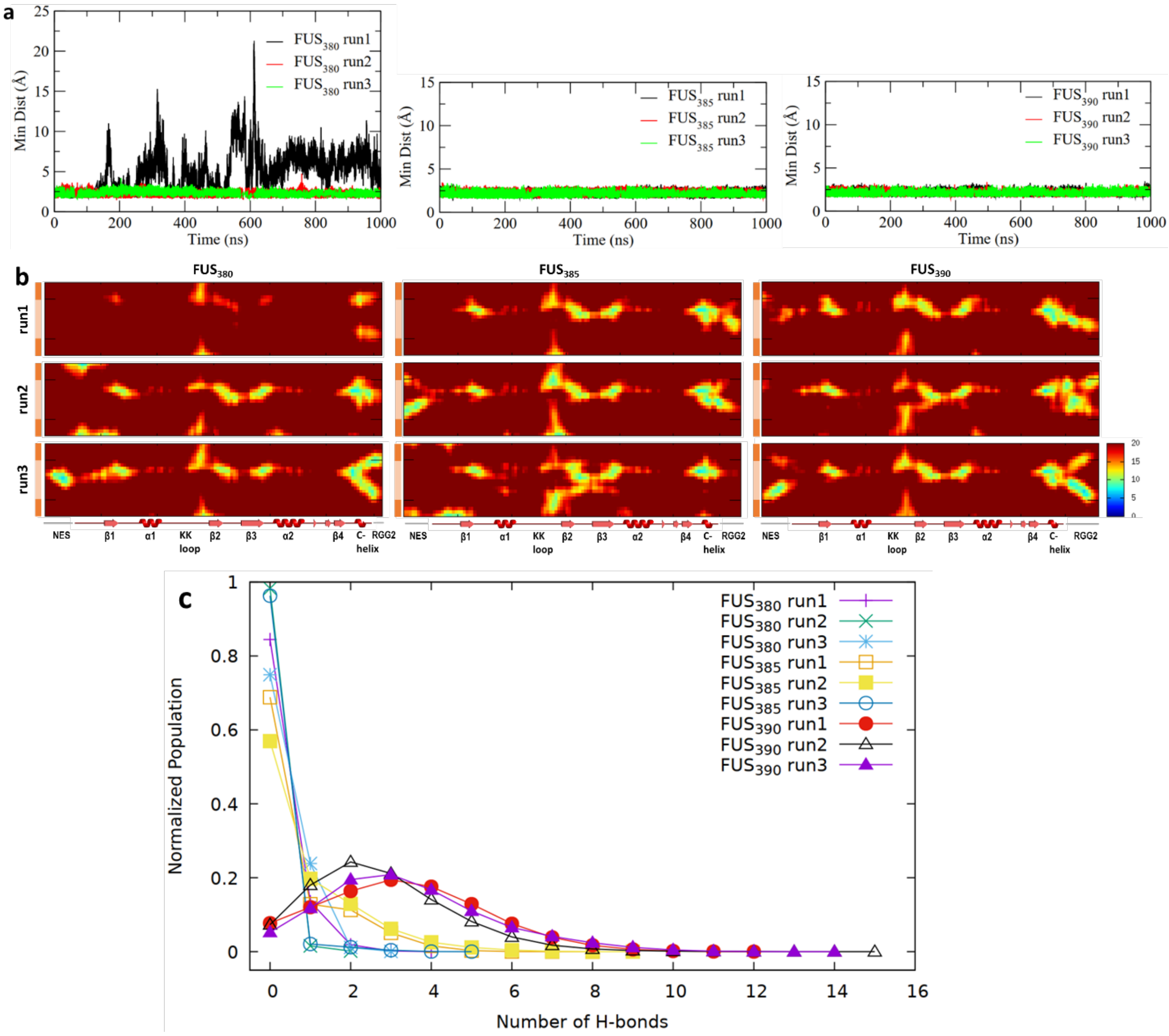

Fig. S4: Dynamics of FUS_{380}, FUS_{385} *and* FUS_{390}. (a) Variation in the minimum distance between the β-sheet surface of RRM (286-290, 322-324, 336-340 AA) and the RNA hairpin plotted for the three independent runs. (b) The inter-atomic distances between the residues of *FUS* (FUS_{380}, FUS_{385} *and* FUS_{390} in columns) and RNA averaged over the last 100 ns simulation of the three replicates (in rows). The secondary structures of FUS are represented on the x-axis, while the RNA stem (dark orange) and RNA loop (light orange) are represented on the y-axis. (c) Population distribution of the number of hydrogen bonds formed between the RGG2 and RNA. The results from three independent runs of FUS_{380}, FUS_{385} and FUS_{390} are shown. It is clear from the distribution that the RNA in FUS_{380} expresses only 2 or less h-bonds, while the RNA in FUS_{385} expresses 3 or less h-bonds. Among the three variants, the FUS_{390} with three RGG repeats shows the maximum number of h-bonds up to 8. This number is consistent in the three independent simulations indicating stable dynamics.

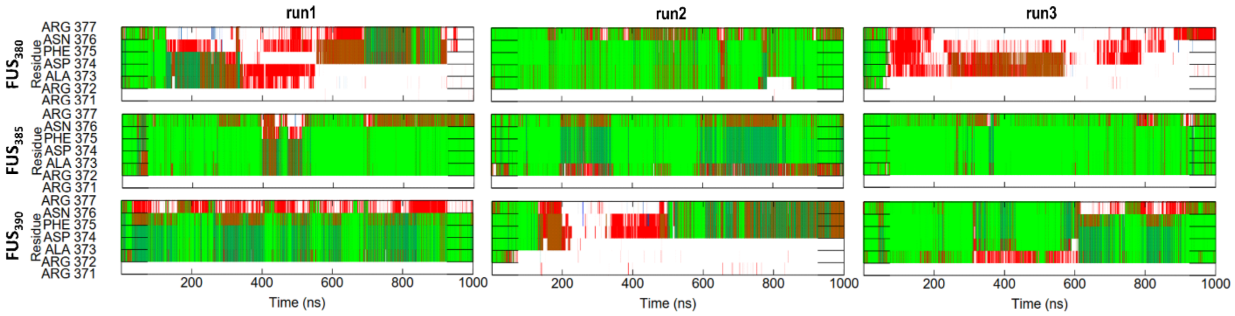

Fig. S5: Secondary structure analysis depicting the stability of the C-terminal helix in FUS_{380}, FUS_{385}, and FUS_{390} systems. Light green: α-helix, Dark green: 3_{10} helix, Red: turns.

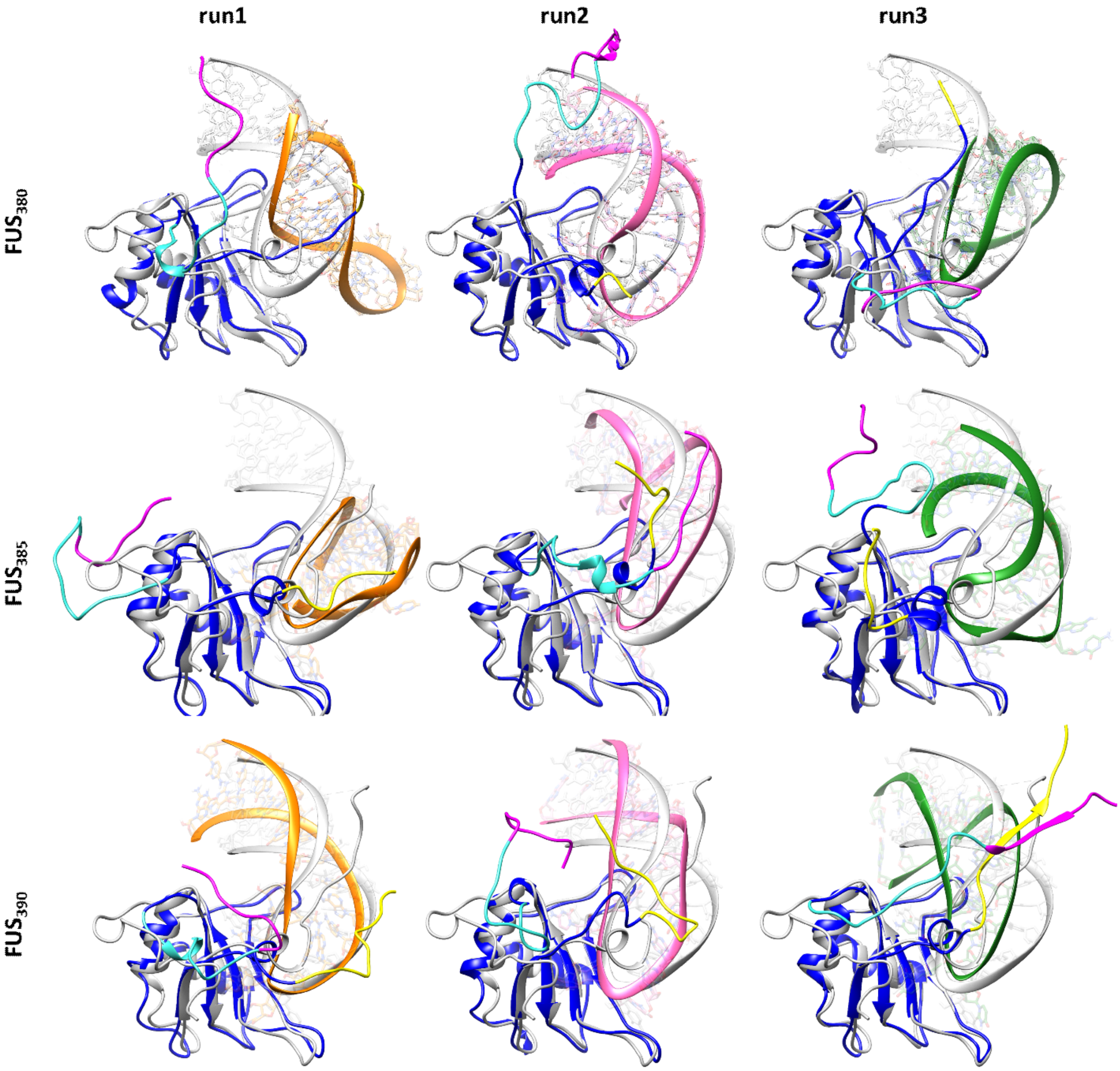

Fig. S6: Structure superposition of NMR (gray) and 1 μs simulated conformations of FUS_{380}, FUS_{385} and FUS_{390}. All conformations sampled during the simulation can be visualized using the trajectories available online. The different regions of FUS in the simulated conformations are colored as RGG1 in magenta, NES in cyan, RRM in blue, and RGG2 in yellow, while the RNA of the three independent simulations are colored in orange, pink and green, respectively. Visually, it is clear that the stability of the RNA binding orientation increases with the addition of RGG repeats.

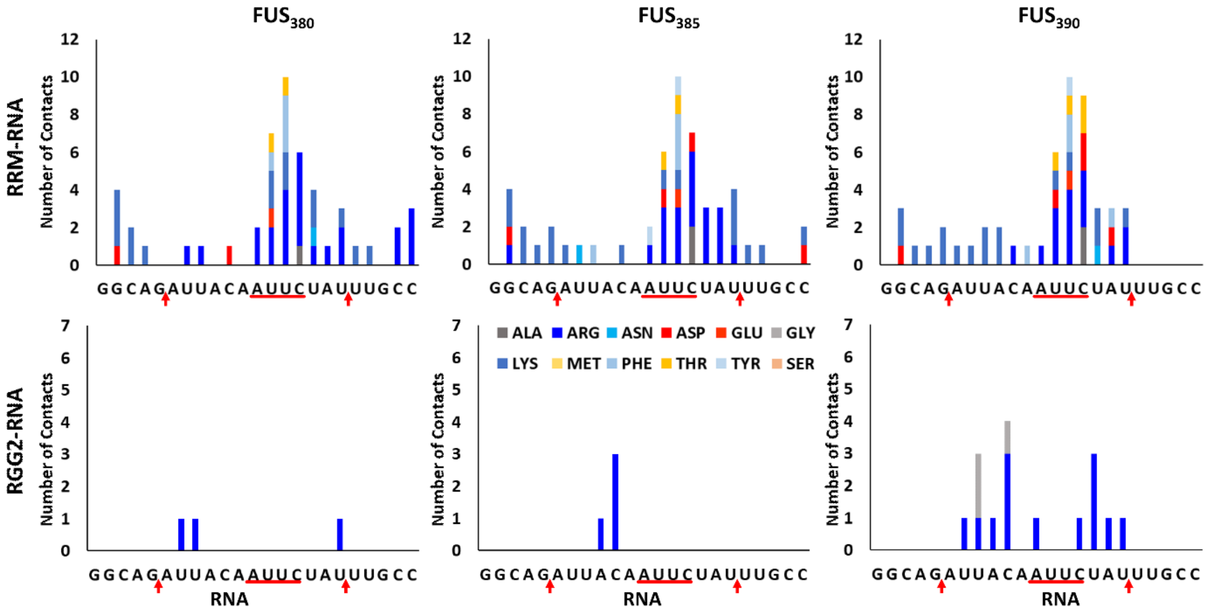

Fig. S7: Amino acid-wise interaction histogram depicting the number of interactions by each amino acid in the RRM and RGG2 domains with the individual bases of the 23mer RNA of *FUS*380, *FUS*385 and *FUS*390. These interactions are calculated as an average over the last 100 ns of all three replicate simulations.

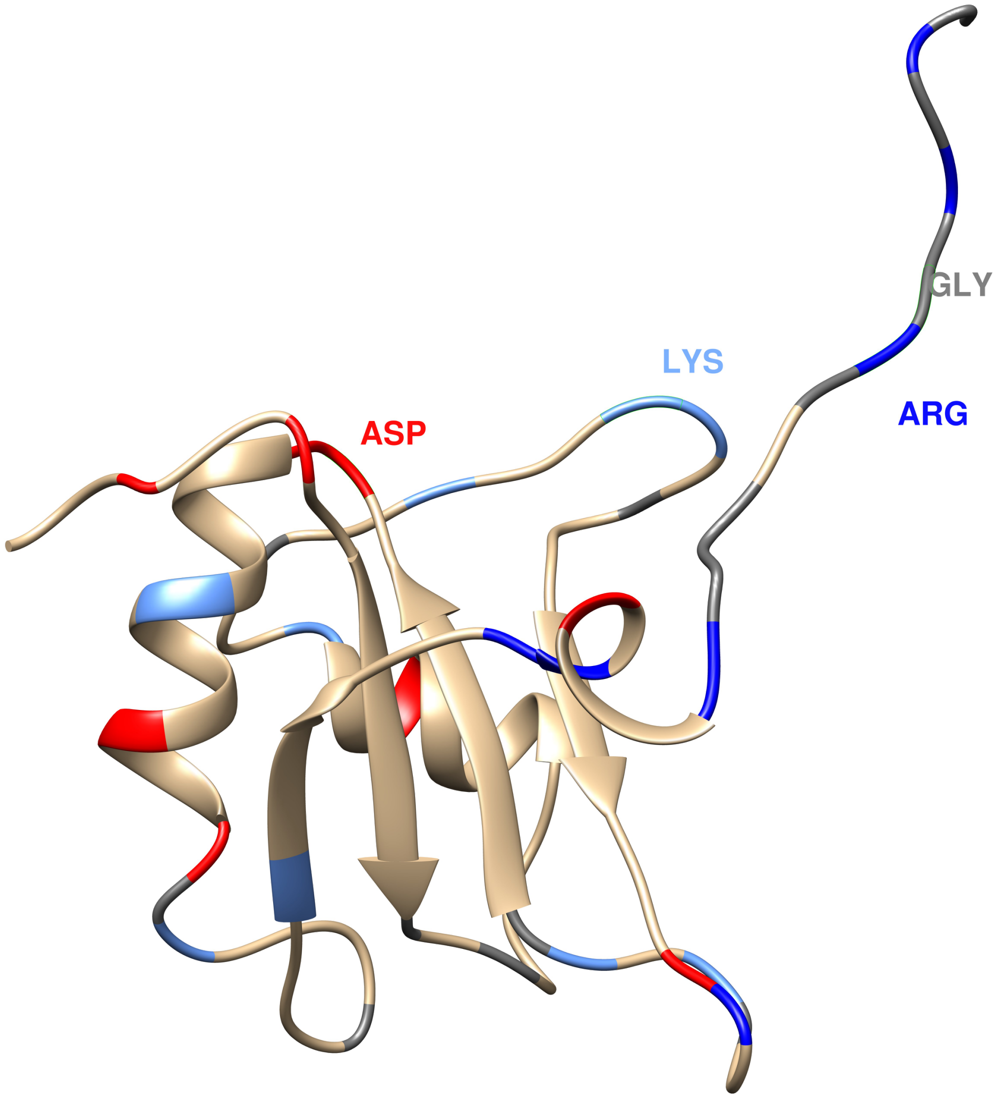

Fig. S8: The location of key residues Arg (blue), Lys (light blue), Asp (red), and Gly (gray) in the three-dimensional structure of RRM and RGG2 is depicted in FUS_{390} structure (PDB ID: 6SNJ).

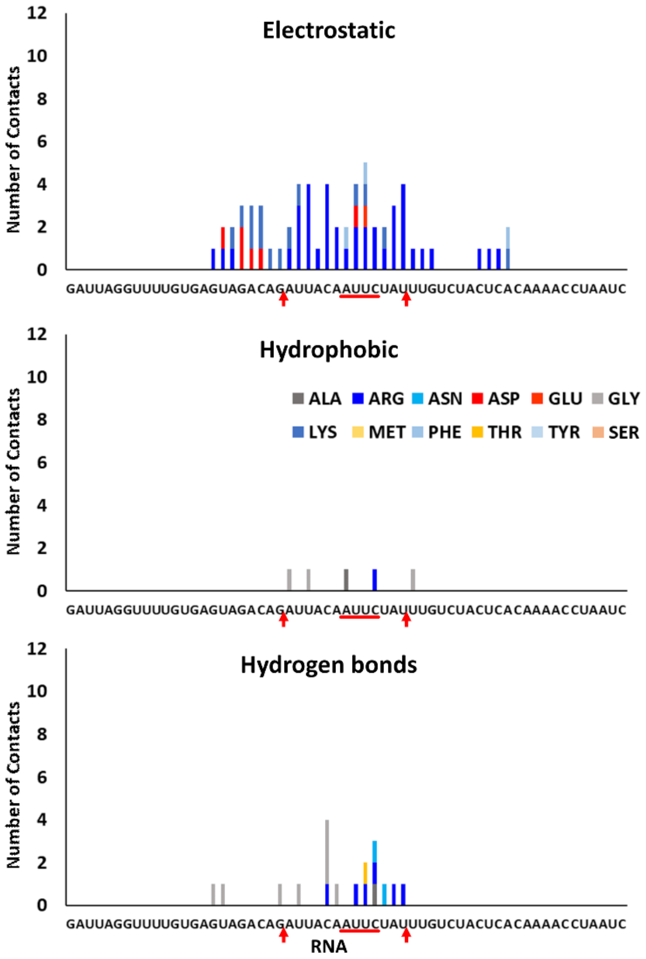

Fig. S9: Amino acid-wise interaction histogram depicting the number of electrostatic, hydrophobic, and hydrogen bond interactions by each amino acid in FUS_{418}. The number of interactions for each amino acid includes both RRM and RGG2.

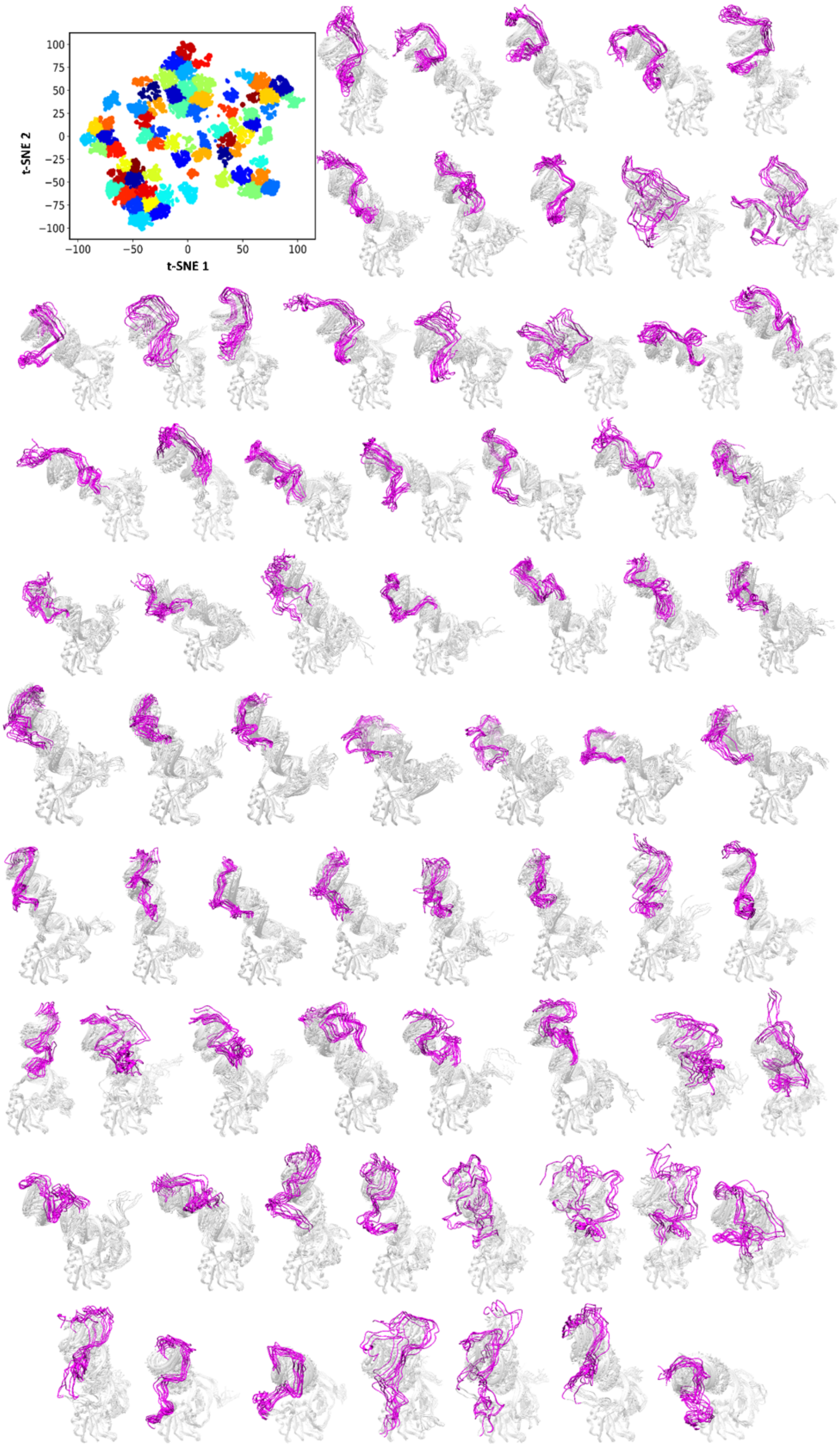

Fig. S10: Clustering of the $FUS_{223-418}$ ensemble by t-SNE and kMeans methods. The projection of the first two tSNE components classifies the sampled conformations into 70 distinct and unique clusters. 10 conformers from each cluster are superimposed and shown. The RRM domain, RGG2 and RNA are shown in gray. The RGG1 is shown in magenta. The entire RGG1 region interacts with the RNA, which is unlike the two distinct sites of interaction shown by RGG2.

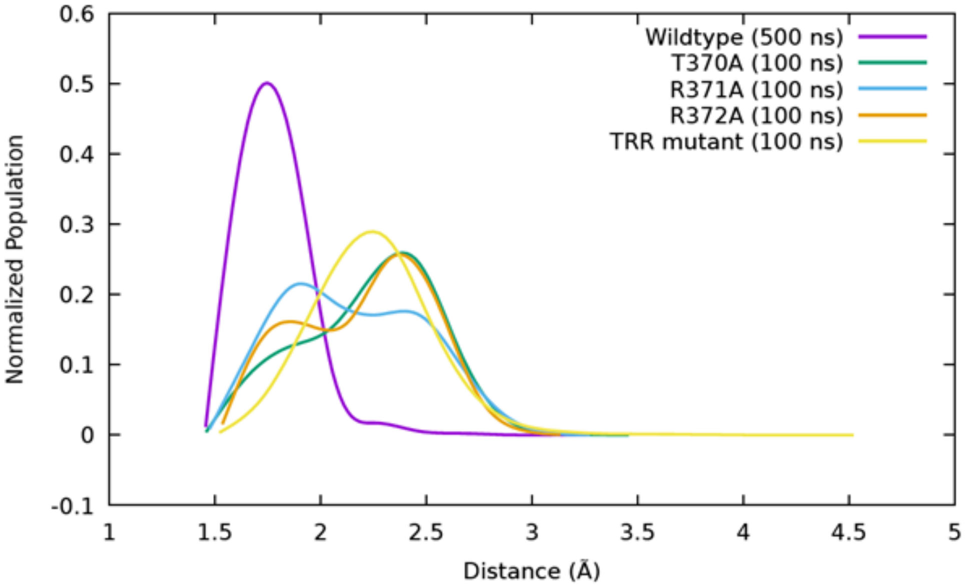

Fig. S11. Population distribution of the minimum distance between the β-sheet surface of RRM (286-290, 322-324, 336-340 AA) and the RNA hairpin in $FUS_{223\text{-}418}$-RNA complexes. In order to explore the importance of the RRM-RGG2 boundary residues (369-377 AA), we identified three important residues (T370, R371, R372) from the FUS_{RRM} simulation and performed Ala mutation studies. The distance between RRM surface and RNA hairpin in the mutant systems is higher than the wildtype and clearly shows fluctuation upto 3 Å. This highlights the functional importance of the boundary residues which are equivalent to the linker regions connecting globular domains. The *in vivo* effects of these mutations are currently being tested.

Force field Limitations: Force fields play a crucial role in the simulation of biomolecules. Further adding to this challenge is the representation of IDPs and IDRs present in mixture with folded domains. Lately, several IDP force field models have appeared in the literature and are being applied (and subjected to evaluation) [41]. A study by Sarthak et al. [33] comparing the behavior and efficiency of several versions of AMBER [42], CHARMM [43] and D.E. Shaw [41, 44] force fields to model the dynamics of FUS has claimed that the a99SB-disp [41] gives the best results. Along with a99SB-disp for FUS, the authors have tested the D.E. Shaw's modifications of ff14 for the RNA [46] since both these force fields were parameterized to use with TIP4P-D water model.

In order to choose the force fields wisely, in a data-driven manner, we performed simulations of the NMR structure 6SNJ using ff14SB+OL3 force field (specific for folded proteins and similar to a study by Pokorna et al., [32] named 14sb_OL3) and a99SB-disp + D.E.Shaw RNA force field (similar to the combination used by Sarthak, K et al., named disp_shaw). The RMSDs of RGG2 (378-390) and RNA (relative to RRM) calculated for the three different systems (including a99SB-disp + OL3, named disp_OL3), shown in Fig. S14 below, clearly highlight the inadequacy of using non-IDP specific force field to model proteins with IDRs. The RMSD of RGG2 does not show any significant changes in the 14sb_OL3 simulation indicating that this region is over-stabilized by ff14SB. This RGG2 region has been speculated to unwind the stem-loop junction of a dsRNA hairpin and make it a single strand to recruit the zinc finger domain. However, the ff14SB highly dampens the dynamics of RGG2 and might prevent the unwinding of the RNA hairpin, thus highlighting the importance of using IDP specific force field to model even a small stretch of RGG.

The RGG2 of disp_shaw and disp_OL3 behave very similarly and samples a larger conformational space than 14sb_OL3. This behavior is expected since FUS is modeled using the a99SB-disp ff in both the systems. However, the internal dynamics of RNA among these simulations are slightly different. The RNA is highly rigid in the disp_shaw simulations (eRMSD <0.75) whereas the RNA in disp_OL3 and 14sb_OL3 are slightly flexible (eRMSD ~1). Yet, the FUS-RNA complex remains stable in all three systems as seen by the RMSD of RNA with respect to the RRM position. Though D.E. Shaw RNA force field has been used by Sarthak et al., the application of OL3 for RNA is widely accepted and time tested. Also, we have observed overstabilization of RNA when using D.E.Shaw force field and hence we proceeded to use the combination of a99SB-disp + OL3 for our simulations.

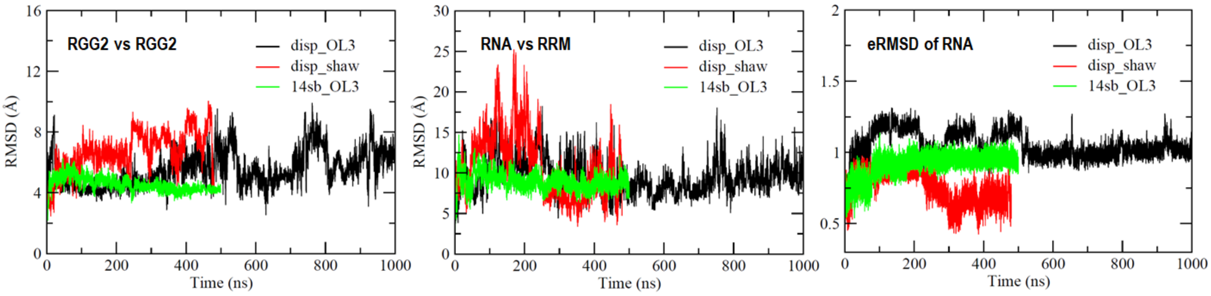

Fig. S12: Effect of force fields on FUS_{390}-RNA complex. The RMSD of RGG2 with RGG2 as reference indicates the conformational dynamics of RGG2 and highlights the importance of using a force field that also accounts for disordered proteins. The force field that are designed for folded proteins only (shown in green) over-stabilizes the RGG2 and in turn dampen all the conformational dynamics. Similarly, the effect of RNA force fields is shown by using OL3 and D.E. Shaw models. The RMSD of RNA with respect to the stable RRM shows the orientational dynamics of RNA and again highlights the importance of using a force field parameterized for disordered proteins. The rigidity of RGG2 in ff14SB force field is transferred to the RNA also and hence the RNA dynamics is dampened in case of 14SB_OL3. When the intramolecular stability of RNA is considered, the eRMSD measure shows both 14SB_OL3 and disp_shaw over stabilizes the RNA. Our analysis reinforces the importance of choosing the right force field for studying specific systems.

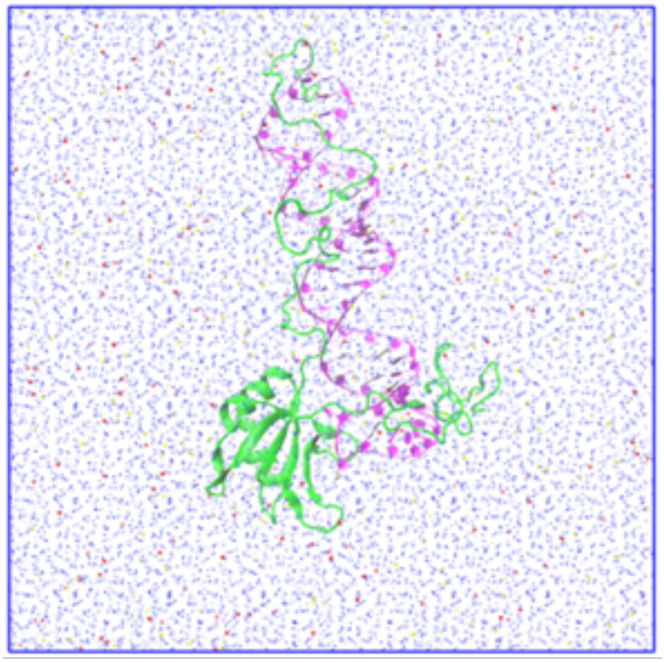

Fig. S13. The solvated $FUS_{223-418}$-RNA complex. The extent of simulation box is shown as blue boundary in which the water, ions and protein-RNA complex are embedded. The Na+ and Cl- ions are shown as purple and green spheres, respectively, the protein in green and RNA in magenta. A cross section of the simulation box with one layer of water molecules (in licorice) is shown.

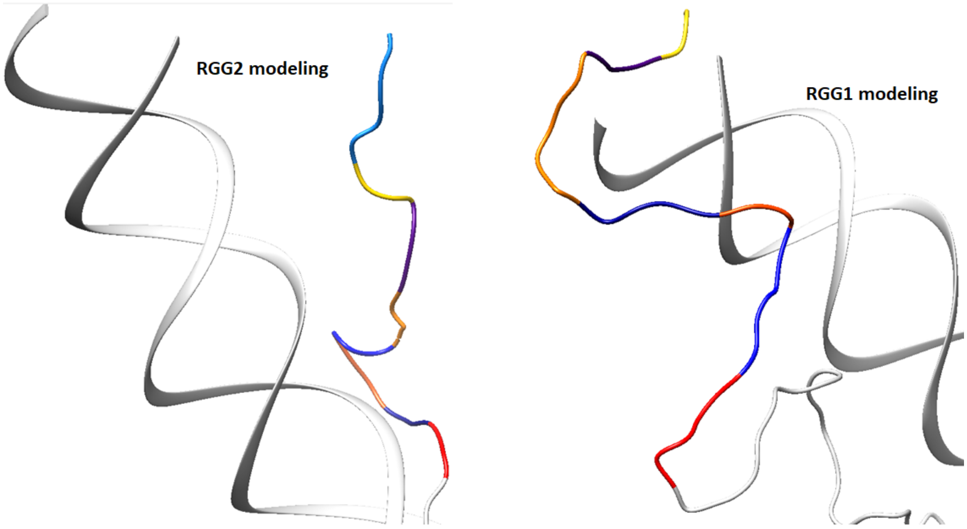

Fig. S14: Modeling of RGGs. The fragments that were added sequentially are shown in different colors.

TABLE S1: Average lifetime of interactions calculated per residue with the AUUC (AAUG in case of RNAmut systems) motif in one of the three simulation trajectories of all the studied systems. The lifetimes were calculated by averaging the lifetimes of all-atom pairs per residue within a distance of 7 Å, normalized by the total number of contacts per residue-base pair.

Systems	A	U/A	U	C/G
6GBM (NMR)	Arg328 (45%)	Thr326 (83%), Arg328 (68%), Lys334 (79%), Asn376 (56%)	Phe288 (91%), Tyr325 (80%) Lys334 (36%), Thr370 (76%), Arg372 (80%)	Asp283 (77%), Asn285 (86%), Thr370 (78%) Arg371 (80%), Arg372 (68%), Ala373 (52%)
6SNJ (NMR)	Thr326 (91%), Arg328 (51%), Arg377 (75%)	Thr326 (90%), Lys334 (84%), Arg372 (82%), Asn376 (76%), Arg377 (81%)	Tyr325 (77%), Thr338 (84%), Arg372 (85%), Ala369 (96%), Phe368 (94%), Asn263 (97%), Lys264 (78%)	Thr370 (88%), Arg371 (83%), Thr286 (93%), Asn284 (83%), Asp283 (56%)
FUS_{RRM}	Arg328 (21%)	Thr326 (23%), Arg328 (27%), Lys334 (20%), Arg372 (1%)	Phe288 (44%), Lys334 (17%), Thr370 (41%)	Asn323 (23%), Tyr325 (25%)
FUS_{380}	Arg328 (11%)			
FUS_{385}	Arg372 (37%)	Arg328 (63%), Lys334 (39%), Arg372 (35%), Thr326 (42%)	Phe288 (66%), Tyr325 (51%), Lys334 (19%), Thr370 (73%), Arg372 (46%), Phe375 (34%)	Thr286 (66%), Tyr325 (33%), Thr338 (36%), Ala369 (55%), Arg371 (72%), Arg372 (38%)
FUS_{390}	Ala373 (32%), Asn376 (22%), Gly380 (23%)	Tyr325 (29%), Arg328 (43%), Arg372 (34%)	Phe288 (61%), Tyr325 (44%), Thr370 (67%), Arg372 (39%)	Thr286 (58%), Thr338 (31%), Ala369 (52%), Arg371 (59%)
FUS_{418}	Ala373 (42%), Asp374 (20%)	Thr326 (36%), Arg328 (65%), Arg372 (44%)	Phe288 (46%), Tyr325 (45%), Thr370 (56%), Arg372 (50%)	Asn284 (34%), Ala369 (41%), Arg371 (73%)
$FUS_{223-418}$	Arg372 (31%), Ala373 (24%)	Tyr325 (63%), Thr326 (46%), Arg328 (56%)	Phe288 (32%), Asn323 (32%), Tyr325 (48%), Thr370 (48%)	Met321 (23%), Arg371 (41%), Arg372 (23%), Ala373 (22%)
$FUS_{418}-RNAmut$	Arg372 (52%), Ala373 (48%),	Arg328 (69%)	Phe288 (51%), Asn323 (24%), Tyr325 (49%), Thr338 (46%), Ala369 (63%), Thr370 (76%)	Ala369 (58%), Arg372 (56%)

$FUS_{390} - RNAmut$	Arg372 (31%)	Lys334 (23%), Arg372 (34%)	Phe288 (61%), Tyr325 (44%), Lys334 (9%), Thr370 (67%), Arg372 (39%)	Asn284 (35%), Ala369 (52%), Thr370 (48%), Arg372 (20%)

Table S2: The description of the FUS systems simulated in this study.

Name	Box size (Å)	Number of atoms	Number of Ions	
			Na+	Cl-
$FUS_{RRM}-core$	75.9 * 75.9 * 75.9	54,296	65	40
FUS_{RRM}	79.9 * 79.9 * 79.9	65,402	69	46
$FUS_{RRM}-KKK_{mut}$	75.9 * 75.9 * 75.9	54,289	66	40
FUS_{380}	79.89 * 79.89 * 79.89	65,288	69	46
FUS_{385}	79.89 * 79.89 * 79.89	65,298	68	46
FUS_{390}	82.1 * 82.1 * 82.1	71,749	70	50
FUS_{418}	130.2 * 130.2 * 130.2	284,638	253	199
$FUS_{223-418}$	127.85 * 127.85 * 127.85	272,692	239	189
$FUS_{390}-RNA_{mut}$	82.1 * 82.1 * 82.1	71,759	70	50
$FUS_{418}-RNA_{mut}$	125.5 * 125.5 * 125.5	257,876	233	179

References (numbered according to the main text):

32. Pokorna, P., Krepl, M., Campagne, S., and Sponer, J. (2022) Conformational Heterogeneity of RNA Stem-Loop Hairpins Bound to FUS-RNA Recognition Motif with Disordered RGG Tail Revealed by Unbiased Molecular Dynamics Simulations. *J. Phys. Chem. B* **126**, 9207–9221

33. Sarthak, K., Winogradoff, D.N., and Aksimentiev, A. (2022) Benchmarking molecular dynamics force fields for simulations of biological condensates. *Biophys. J.* **121**, 197a
Robustelli, P., Piana, S., and Shaw, D.E. (2018) Developing a molecular dynamics force field for both folded and disordered protein states. Proc. Natl. Acad. Sci. U. S. A. 115, E4758--E4766

42. Maier, J.A., Martinez, C., Kasavajhala, K., Wickstrom, L., Hauser, K.E., and Simmerling, C. (2015) ff14SB: Improving the Accuracy of Protein Side Chain and Backbone Parameters from ff99SB. J. Chem. Theory Comput. 11, 3696–3713

43. Huang, J., Rauscher, S., Nawrocki, G., Ran, T., Feig, M., De Groot, B.L., Grubmüller, H., and MacKerell, A.D. (2016) CHARMM36m: An improved force field for folded and intrinsically disordered proteins. Nat. Methods 14, 71–73

44. Piana, S., Robustelli, P., Tan, D., Chen, S., and Shaw, D.E. (2020) Development of a Force Field for the Simulation of Single-Chain Proteins and Protein-Protein Complexes. J. Chem. Theory Comput. 16, 2494–2507

46. Tan, D., Piana, S., Dirks, R.M., and Shaw, D.E. (2018) RNA force field with accuracy comparable to state-of-the-art protein force fields. Proc. Natl. Acad. Sci. U. S. A. 115, E1346–E1355

67. Laskowski, R.A., Hutchinson, E.G., Michie, A.D., Wallace, A.C., Jones, M.L., Thornton, J.M. (1997) PDBsum: A Web-based database of summaries and analyses of all PDB structures. *Trends Biochem. Sci.* **22,** 488-490
